# PEX19 restricts porcine deltacoronavirus replication through farnesylation-dependent and -independent mechanisms

**DOI:** 10.1128/jvi.02097-25

**Published:** 2026-03-24

**Authors:** Chaoqun Chen, Guanning Su, Yuchen Wang, Yuanxiang Xiong, Wenwen Xiao, Zhuang Li, Liurong Fang, Yanrong Zhou, Shaobo Xiao

**Affiliations:** 1 National Key Laboratory of Agricultural Microbiology, College of Veterinary Medicine, Huazhong Agricultural University 627716, Wuhan, China; 2 Key Laboratory of Preventive Veterinary Medicine in Hubei Province, Cooperative Innovation Center for Sustainable Pig Production, Wuhan, China; University of Michigan Medical School, Ann Arbor, Michigan, USA

**Keywords:** porcine deltacoronavirus (PDCoV), peroxisome biogenesis factor 19 (PEX19), farnesylation modification, antiviral mechanism, innate immunity

## Abstract

Peroxisomes are vital cellular organelles that play critical roles in metabolism, immune regulation, and disease pathogenesis. As a key receptor for peroxisomal membrane proteins, peroxisomal biogenesis factor 19 (PEX19) is essential for peroxisome biogenesis. In this study, we identify PEX19 as a novel host restriction factor against porcine deltacoronavirus (PDCoV), an emerging enteropathogenic coronavirus with zoonotic potential. Overexpression of PEX19 significantly inhibits PDCoV replication, while knockout of PEX19 enhances viral propagation. Interestingly, the anti-PDCoV effect of PEX19 largely depends on its farnesylation modification, as PEX19 mutants with deleted or mutated farnesylation sites exhibit only marginal anti-PDCoV activity. Mechanistically, PEX19 restricts PDCoV infection through three distinct pathways: (i) reducing cellular cholesterol levels in a farnesylation-dependent manner, (ii) targeting the viral nonstructural protein 2 (nsp2) for autophagy-lysosome-mediated degradation, which is also dependent on farnesylation, and (iii) inducing low-level interferon production independently of farnesylation. Taken together, these findings define a new antiviral role for PEX19 and highlight its potential as a therapeutic target for combating PDCoV infection.

**IMPORTANCE:**

Peroxisomes are increasingly recognized as critical regulators of virus-host interactions; however, their roles during coronavirus infection remain poorly understood and controversial. By screening the peroxins (PEXs) that regulate the replication of porcine deltacoronavirus (PDCoV), we identify PEX19, a key peroxisomal biogenesis factor, as a novel antiviral host protein, whose anti-PDCoV activity is largely dependent on farnesylation modification. Our findings demonstrate that farnesylated PEX19 restricts PDCoV replication by reducing cellular cholesterol levels and promoting autophagy-lysosome-mediated degradation of the viral nsp2 protein, while also inducing low-level interferon production independently of farnesylation. These results provide new molecular insights into PDCoV-host interactions and highlight PEX19 as a potential therapeutic target against PDCoV infection.

## INTRODUCTION

Peroxisomes are essential metabolic organelles that perform various functions, including the α- and β-oxidation of fatty acids, cholesterol metabolism, the biosynthesis of bile acids and ether phospholipids, immunometabolism, the regulation of reactive oxygen species (ROS), and antiviral responses (1–4). Their biogenesis relies on a group of proteins known as peroxins (PEXs), with over 30 members identified across different species. In humans, mutations in several PEXs are associated with severe peroxisome biogenesis disorders (PBDs) (5). Based on their biological roles, PEXs are broadly categorized into groups responsible for peroxisomal membrane assembly, matrix protein import, and organelle proliferation.

The formation of the peroxisomal membrane is the initial step in peroxisome biogenesis and involves the integration and assembly of multiple membrane proteins (6, 7). Key players in this process include PEX3, PEX16, and PEX19 (8). Since peroxisomes lack their own genetic material, all peroxisomal membrane and matrix proteins are encoded by nuclear genes, synthesized in the cytosol, and subsequently imported into the organelle (9). Proteins such as PEX5 and PEX7 are essential for the import of matrix proteins (10). Finally, peroxisomes can proliferate by growth and division of preexisting peroxisomes, a process in which members of the PEX11 family play pivotal roles (11, 12).

PEX19 is a core regulatory protein involved in the transport of peroxisomal membrane proteins (PMPs) (13, 14). As a chaperone, PEX19 binds newly synthesized PMPs in the cytosol to prevent their misfolding or aggregation (15). In addition, PEX19 serves as a shuttle receptor, recognizing newly synthesized PMPs and delivering them to the peroxisomal membrane for insertion (16). PEX19 also directly participates in membrane insertion, ensuring the proper integration of PMPs into the peroxisomal membrane (17, 18). Notably, PEX19 plays an essential role in early steps of peroxisome biogenesis (19, 20); for example, the transport of PEX3 from the endoplasmic reticulum (ER) to peroxisomes requires functional PEX19 (21).

PEX19 undergoes a post-translational modification known as farnesylation, which is highly conserved throughout evolution except in trypanosomes (22). Farnesylation, catalyzed by farnesyltransferase (FTase), is an irreversible modification that covalently attaches a C15 isoprenoid group to the cysteine residue within the C-terminal CaaX motif of PEX19 (23, 24). This modification is critical for the function of PEX19. In *Saccharomyces cerevisiae*, farnesylated PEX19 displays a 10-fold higher affinity for PMPs compared to its non-farnesylated form, and yeast cells lacking PEX19 farnesylation exhibit PMP instability and defects in peroxisome biogenesis (25). It is proposed that farnesylation may enhance PEX19 function by inducing conformational changes, although the precise molecular mechanisms and functional consequences remain to be fully elucidated.

To counteract host antiviral defenses, a number of viruses have evolved strategies to manipulate peroxisome biogenesis, function, or signaling. Human cytomegalovirus (HCMV) infection has been shown to increase peroxisome abundance, enhancing lipid metabolism to facilitate viral assembly and release (26, 27). In contrast, flaviviruses such as Zika virus (ZIKV) and dengue virus (DENV) inhibit peroxisome biogenesis by targeting key biogenesis factors, including PEX19, thereby dampening the peroxisome-dependent interferon (IFN) response and promoting viral replication (28). Similarly, coronaviruses have been reported to modulate peroxisome dynamics, although findings are inconsistent. SARS-CoV-2 infection was shown to transiently increase peroxisome abundance and redistribute them toward viral replication sites to potentially protect viral RNA from oxidative damage (29). Conversely, other studies reported a marked reduction in peroxisome numbers following infection, mediated by viral proteins such as ORF9c that impair peroxisomal protein import, ultimately compromising peroxisomal function (30).

Porcine deltacoronavirus (PDCoV) is an emerging enteropathogenic coronavirus that causes diarrhea, vomiting, dehydration, and death in nursing piglets (31–33). PDCoV has garnered more attention after a study that reported the detection and isolation of PDCoV from plasma samples of three Haitian children with acute undifferentiated febrile illness (34). As a result, PDCoV has been proposed as the eighth coronavirus capable of infecting humans (35). Despite extensive studies on PDCoV pathogenesis, the interplay between PDCoV and host peroxisomes has not yet been explored.

In this study, we aimed to investigate the role of PEXs during PDCoV infection, with a particular focus on elucidating the specific mechanism by which PEX19 regulates PDCoV replication. Our findings provide new insights into the molecular basis of PDCoV-host interactions and present a novel potential target for the development of antiviral strategies against PDCoV.

## RESULTS

### PEX19 exhibits stronger anti-PDCoV activity

**Fig 1:**
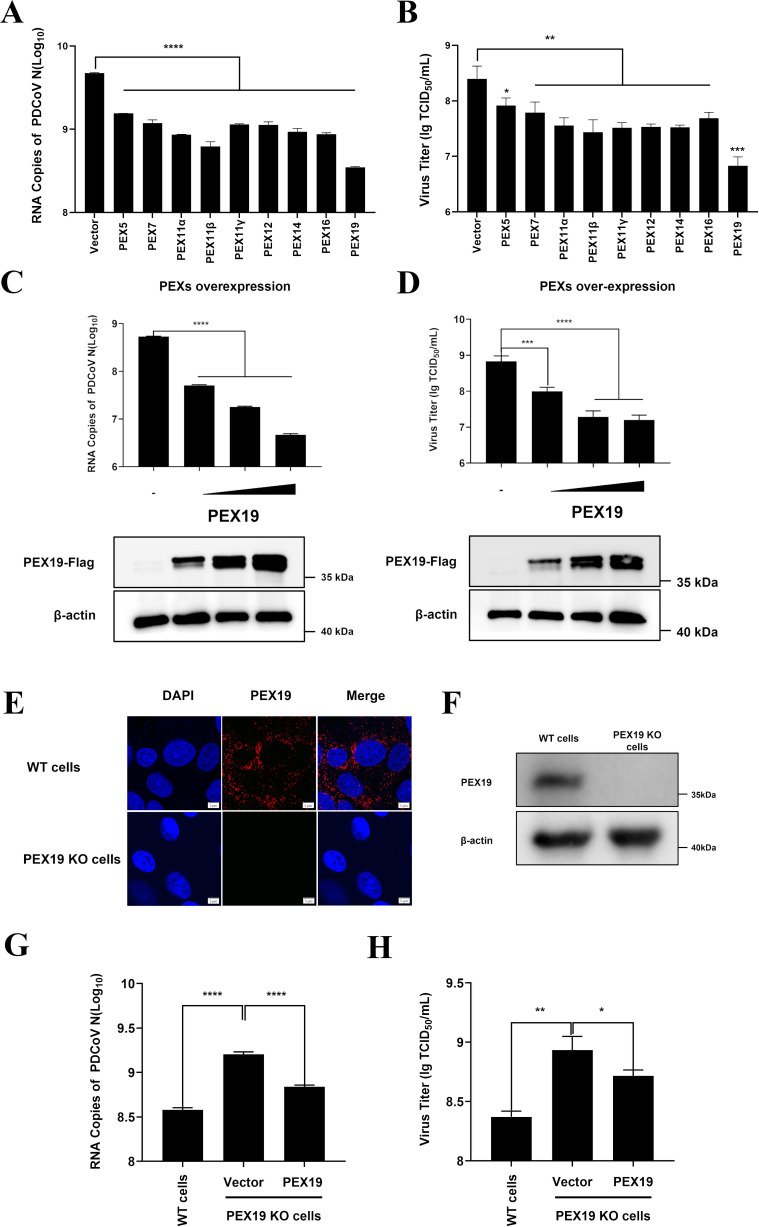
PEX19 exhibits stronger anti-PDCoV activity. (**A and B**) LLC-PK1 cells were transfected with plasmids encoding PEX5, PEX7, PEX11α, PEX11β, PEX11γ, PEX12, PEX14, PEX16, PEX19, or an empty vector as control. At 12 h post-transfection, cells were infected with PDCoV at a multiplicity of infection (MOI) of 1. At 12 h post-infection, cell lysates and supernatants were harvested to assess viral replication by RT-qPCR (**A**) and TCID₅₀ assay (**B**). (**C and D**) Dose-dependent inhibition of PDCoV replication by PEX19. LLC-PK1 cells were transfected with increasing amounts (0, 0.5, 1.0, and 2.0 μg) of PEX19 expression plasmid. At 12 h post-transfection, cells were infected with PDCoV (MOI=1). Samples were harvested at 12 h post-infection for viral RNA quantification by RT-qPCR (**C**) and viral titration by TCID_50_ assay (**D**). (**E and F**) Generation and validation of PEX19 knockout (KO) cells. PEX19 expression was analyzed by immunofluorescence assay (scale bar, 5 μm). (**E**) and Western blotting (**F**) using a rabbit polyclonal antibody against PEX19. (**G and H**) Effects of PEX19 knockout and complementation on PDCoV replication. PEX19 KO cells were transfected with either the PEX19 expression plasmid or the empty vector. WT LLC-PK1 cells, which were transfected with an empty vector, were used as controls. At 12 h post-transfection, cells were infected with PDCoV (MOI = 1), and samples were collected 12 h later for RT-qPCR (**G**) and TCID_50_ assay (**H**). Values are shown as means ± SD. Statistical analyses for panels A to D, G, and H were performed with one-way ANOVA. *, *P* < 0.05; **, *P* < 0.01; ***, *P* < 0.001; ****, *P* < 0.0001.

To screen PEXs regulating PDCoV replication, nine key PEXs involved in peroxisome biogenesis, including PEX5, PEX7, PEX11α, PEX11β, PEX11γ, PEX12, PEX14, PEX16, and PEX19, were chosen, and the eukaryotic expression plasmids encoding these PEX proteins were constructed. Subsequently, LLC-PK1 cells were transfected with each of these eukaryotic expression plasmids, followed by infection with PDCoV at a multiplicity of infection (MOI) of 1. Expression of the PEX proteins was confirmed by Western blot analysis (Fig. S1A). Viral replication was assessed by RT-qPCR (Fig. 1A; Fig. S1B) and TCID_50_ assays (Fig. 1B) at 12 h post-infection (hpi). The results showed that overexpression of each PEX significantly inhibited PDCoV replication compared to the empty vector control, with PEX19 exhibiting the strongest antiviral effect. Moreover, the inhibitory effect of PEX19 on PDCoV replication was in a dose-dependent manner (Fig. 1C and D).

To further validate the anti-PDCoV effect of PEX19, we generated a PEX19 knockout (PEX19 KO) cell line using CRISPR-Cas9 gene editing technology and confirmed the effective knockout of PEX19 protein through immunofluorescence assay (IFA) and Western blot analysis (Fig. 1E and F). Furthermore, no significant difference in cell viability was observed between the PEX19 KO and wild-type (WT) cells (Fig. S1C). Subsequently, PEX19 KO cells were transfected with PEX19 eukaryotic expression plasmid (pCAGGS-Flag-PEX19) and empty vector plasmid, respectively, and then infected with PDCoV (MOI = 1). The WT LLC-PK1 cells transfected with an empty vector served as a control. The results showed that, compared to WT cells, PEX19 knockout significantly enhanced PDCoV replication. However, re-expression of PEX19 in PEX19 KO cells significantly reduced both PDCoV RNA copies and viral titers (Fig. 1G and H). These results collectively demonstrate that PEX19 negatively regulates PDCoV replication.

### The anti-PDCoV effect of PEX19 is associated with its farnesylation

**Fig 2:**
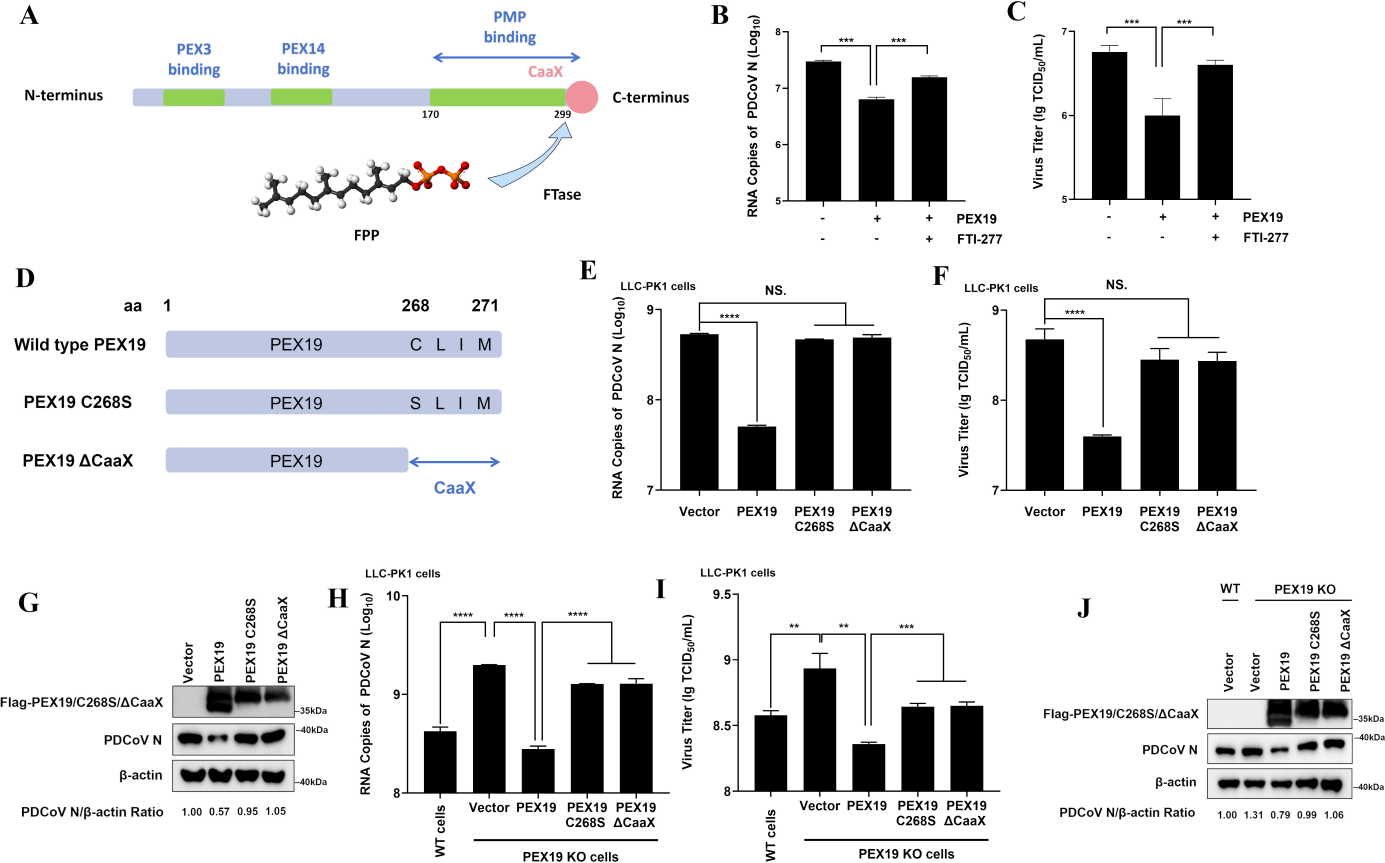
Farnesylation of PEX19 is required for its antiviral activity against PDCoV. (**A**) Schematic diagram of PEX19 structure and farnesylation. (**B and C**) FTI-277 inhibits the antiviral activity of PEX19. Cells were transfected with a PEX19-expressing plasmid or vector control. At 12 h post-transfection, cells were infected with PDCoV at an MOI of 1. At 6 h post-infection (hpi), the culture medium was replaced with fresh medium containing 20 μM FTI-277 or DMSO (vehicle control), and incubation continued for an additional 6 h. Samples were collected at 12 hpi and analyzed by RT-qPCR (**B**) and TCID_50_ assay (**C**). (**D**) Construction of PEX19 farnesylation-deficient mutants. Two PEX19 mutants were generated via site-directed mutagenesis: PEX19 C268S (cysteine at position 268 mutated to serine) and PEX19 ΔCaaX (deletion of the CaaX motif). (**E–G**) Effects of WT and mutant PEX19 on PDCoV replication in LLC-PK1 cells. Cells were transfected with plasmids encoding WT PEX19, PEX19 C268S, or PEX19 ΔCaaX. At 12 h post-transfection, cells were infected with PDCoV (MOI=1). Samples were collected after 12 h post-infection and analyzed by RT-qPCR (**E**), TCID_50_ assay (**F**), and Western blot analysis (**G**). Cells transfected with an empty vector were used as negative controls. (**H–J**) Effects of PEX19 WT and mutants on PDCoV replication in PEX19 KO cells. PEX19 KO cells were transfected with plasmids expressing WT or mutant PEX19. At 12 h post-transfection, cells were infected with PDCoV (MOI = 1) and harvested 12 h later for RT-qPCR (**H**), TCID_50_ assay (**I**), and Western blot analysis (**J**). WT LLC-PK1 cells served as a control. β-actin was used as a loading control. Values are shown as means ± SD. Statistical analyses for panels B, C, E, F, H, and I were performed with one-way ANOVA. NS., no significance; **, *P* < 0.01; ***, *P* < 0.001; ****, *P* < 0.0001.

PEX19 is a hydrophilic farnesylated protein whose post-translational modification process is catalyzed by farnesyltransferase (FTase) (36). This enzyme covalently attaches farnesyl pyrophosphate (FPP) to the cysteine residue in the C-terminal CaaX motif of PEX19 (Fig. 2A). To investigate whether the anti-PDCoV effect of PEX19 is dependent on its farnesylation, we treated cells with FTI-277, a general FTase inhibitor, followed by PDCoV infection. The results showed that FTI-277 treatment significantly attenuated the antiviral activity of PEX19, as evidenced by increased viral RNA levels and elevated viral titers (Fig. 2B and C).

We next constructed PEX19 farnesylation-deficient mutants to further assess the contribution of farnesylation to its antiviral activity. PEX19 is highly conserved among mammalian species, particularly the C-terminal CaaX farnesylation motif (Fig. S2A), underscoring its likely functional importance. Two mutants were constructed using site-directed mutagenesis: PEX19 C268S (where the cysteine at position 268 is mutated to serine) and PEX19 ΔCaaX (which deletes the entire CaaX sequence) (Fig. 2D). The plasmids expressing WT PEX19, PEX19 C268S, and PEX19 ΔCaaX were transfected into LLC-PK1 cells, respectively, and then infected with PDCoV (MOI = 1). The results of RT-qPCR (Fig. 2E), TCID_50_ (Fig. 2F), and Western blot analysis (Fig. 2G) showed that, compared to the empty vector control, WT PEX19 significantly inhibited PDCoV replication, whereas the PEX19 C268S and PEX19 ΔCaaX mutants barely inhibited PDCoV replication. Consistent results were obtained in porcine intestinal epithelial cells (IPI-2I cells) (Fig. S2B and C), indicating that farnesylation modification is crucial for the anti-PDCoV effect of PEX19.

To further validate this finding, PEX19 KO cells were transfected with expression plasmids encoding WT PEX19, PEX19 C268S, or PEX19 ΔCaaX, followed by PDCoV infection (MOI = 1). The results showed that although both WT PEX19 and its farnesylation-deficient mutants (C268S and ΔCaaX) inhibited PDCoV replication in PEX19 KO cells, the inhibitory effects of C268S and ΔCaaX were significantly weaker than those of WT PEX19 (Fig. 2H through J). Collectively, these results further confirm that the anti-PDCoV function of PEX19 is largely dependent on its C-terminal farnesylation modification.

### PEX19 downregulates cholesterol levels in cells through its farnesylation

**Fig 3:**
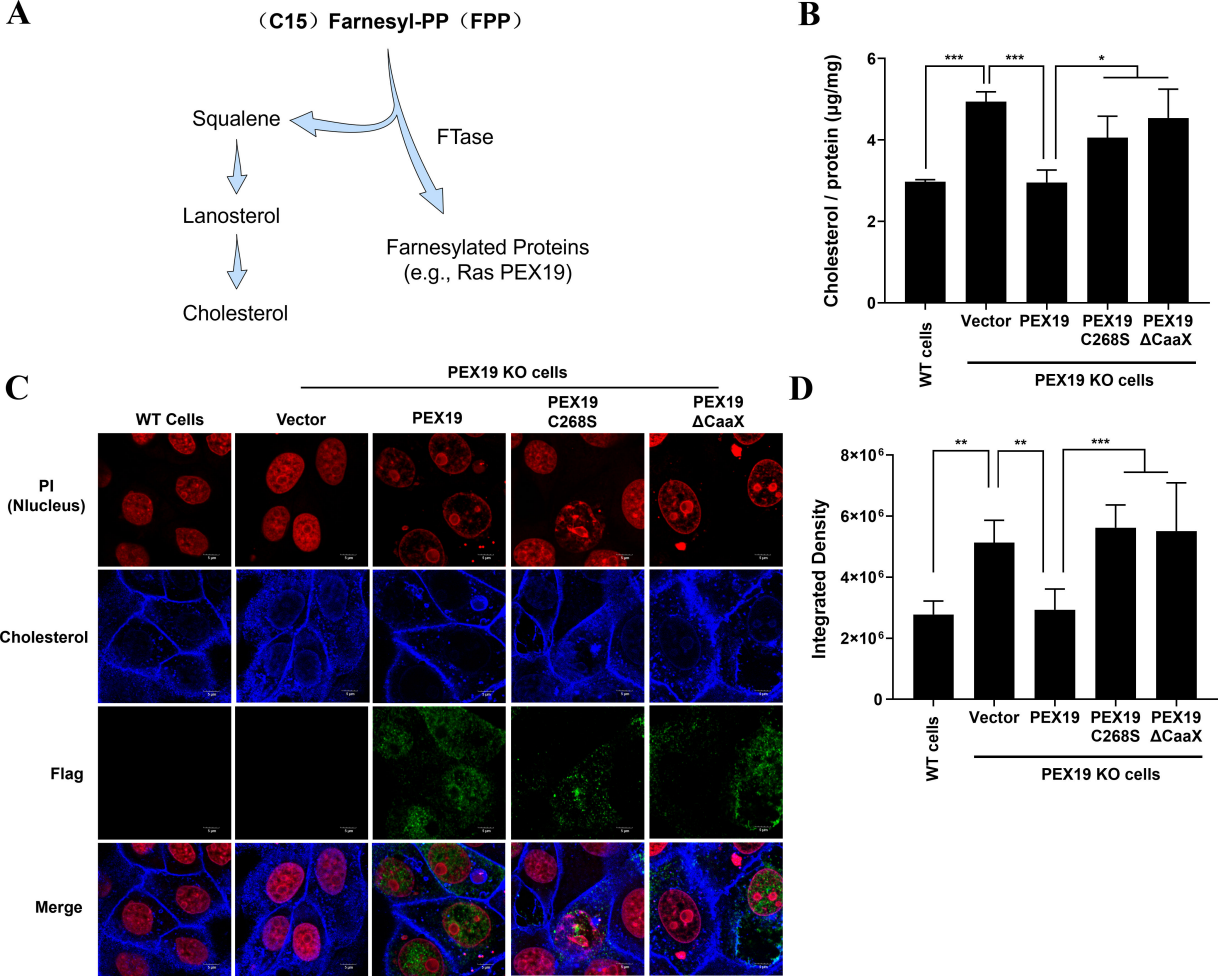
PEX19 negatively regulates cellular cholesterol levels through farnesylation. (**A**) Schematic illustration of the role of farnesyl pyrophosphate (FPP) in cholesterol biosynthesis and protein farnesylation. FPP is synthesized via the mevalonate pathway and serves as a substrate for both squalene synthase in cholesterol biosynthesis and farnesyltransferase (FTase) in protein farnesylation. (**B and C**) Quantification and visualization of cholesterol levels in PEX19 KO cells expressing WT or mutant PEX19. PEX19 KO cells were transfected with plasmids encoding WT PEX19, PEX19 C268S, or PEX19 ΔCaaX. At 24 h post-transfection, cellular cholesterol levels were measured using the Amplex Red Cholesterol Assay Kit (**B**) or visualized by staining with filipin (blue) (**C**). Cell nuclei were counterstained with propidium iodide (PI; red). Fluorescent images were acquired by confocal laser scanning microscopy. Scale bar, 20 μm. (**D**) Quantification of cellular cholesterol levels. Quantification of the cholesterol levels using Image *J* software based on fluorescence intensity measurements from the corresponding microscopic images. The data represent the relative cholesterol content normalized to the control group. Values are shown as means ± SD. Statistical analyses for panels B and D were performed with one-way ANOVA. *, *P* < 0.05; **, *P* < 0.01; ***, *P* < 0.001.

Peroxisomes serve as central organelles for lipid metabolism, and their dysfunction has been closely linked to cholesterol metabolic disorders (37, 38). Farnesylation is a lipid-dependent post-translational modification in which FPP, synthesized via the mevalonate pathway, serves as a substrate (39, 40). As a key metabolic intermediate, FPP can be utilized either by squalene synthase to participate in cholesterol biosynthesis or by FTase to mediate protein farnesylation (Fig. 3A). To investigate whether PEX19 farnesylation is involved in regulating cholesterol metabolism, we reintroduced WT PEX19, PEX19 C268S, or PEX19 ΔCaaX into PEX19 KO cells, respectively, and quantified intracellular cholesterol levels using the Amplex Red Cholesterol Assay Kit at 24 h post-transfection. Compared to WT LLC-PK1 cells, PEX19 KO cells exhibited a significant increase (~67%) in cholesterol content. Reintroduction of WT PEX19 reduced cholesterol levels by approximately 40%, whereas PEX19 C268S and PEX19 ΔCaaX mutants only decreased cholesterol levels by ~18% and ~8%, respectively (Fig. 3B).

To further validate these findings, we performed Filipin staining to visualize cholesterol distribution and analyzed fluorescence intensity using confocal microscopy. As shown in Fig. 3C, PEX19 KO cells displayed a marked increase in cholesterol-associated fluorescence (indicated by the blue signal), whereas the reintroduction of WT PEX19 resulted in a reduction of fluorescence intensity. In contrast, the reintroduction of farnesylation-deficient mutants (PEX19 C268S and PEX19 ΔCaaX) failed to restore cholesterol accumulation (Fig. 3C and D). Collectively, these results indicate that PEX19 plays a crucial role in maintaining cellular cholesterol homeostasis, a function dependent on its farnesylation modification.

### Cholesterol deficiency in cells inhibits PDCoV replication

**Fig 4:**
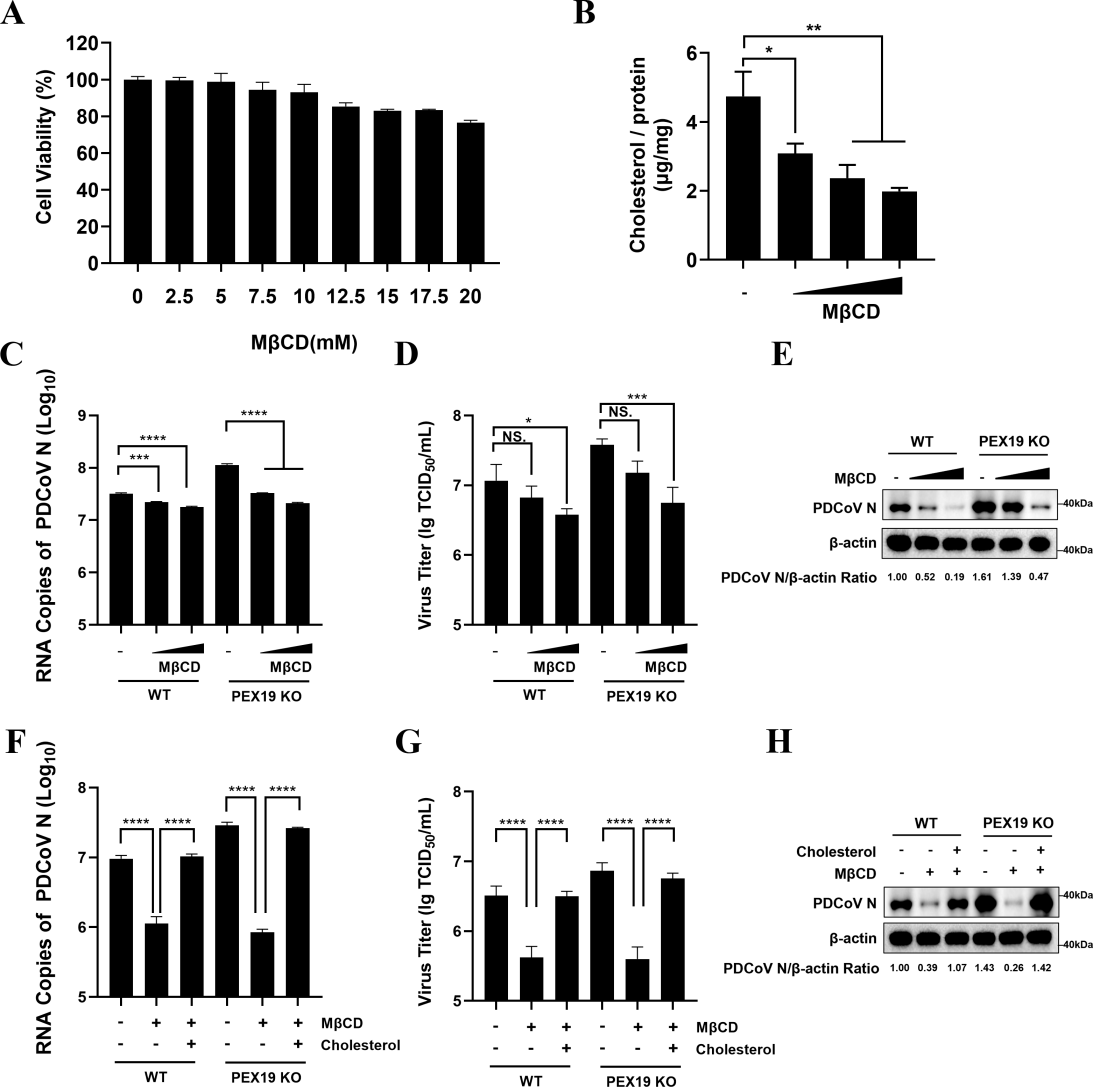
Cholesterol positively regulates PDCoV replication. (**A**) Cytotoxicity of methyl-β-cyclodextrin (MβCD) on LLC-PK1 cells was evaluated using the CCK-8 assay. Cells were treated with increasing concentrations of MβCD (0, 2.5, 5, 7.5, 10, 12.5, 15, 17.5, and 20 mM) for 24 h prior to analysis. (**B**) Cholesterol depletion by MβCD was assessed using the Amplex Red Cholesterol Assay Kit. LLC-PK1 cells were incubated with 0, 5, 10, or 15 mM MβCD for 1 h, and total cholesterol levels were subsequently measured. (**C–E**) Effects of cholesterol depletion on PDCoV replication in PEX19 KO cells. WT and PEX19 KO cells were treated with the indicated concentrations of MβCD (0, 5, 10, or 15 mM) at 37°C for 1 h, followed by infection with PDCoV at an MOI of 1. At 12 h post-infection, viral replication was assessed by RT-qPCR (**C**), TCID₅₀ assay (**D**), and Western blot analysis (**E**). (**F–H**) Restoration of cholesterol rescues PDCoV replication. WT and PEX19 KO cells were pretreated with 10 mM MβCD at 37°C for 1 h, followed by supplementation with water-soluble cholesterol (400 μg/mL) for 1 h. Cells were then infected with PDCoV (MOI = 1), and samples were collected at 12 h post-infection for RT-qPCR (**F**), TCID₅₀ assay (**G**), and Western blot analysis (**H**). β-actin was used as a loading control. Values are shown as means ± SD. Statistical analyses for panels B, C, D, F, and G were performed with one-way ANOVA. NS., no significance; *, *P* < 0.05; **, *P* < 0.01; ***, *P* < 0.001; ****, *P* < 0.0001.

To further investigate how cholesterol affects PDCoV replication, we performed functional validation experiments using the cholesterol-depleting agent methyl-β-cyclodextrin (MβCD) and exogenous water-soluble cholesterol. No evident cytotoxicity could be detected in LLC-PK1 cells when the concentrations of MβCD were ranged within 0–15 mM (Fig. 4A). Thus, we selected MβCD concentrations of 0 mM, 5 mM, 10 mM, and 15 mM for further experiments. Quantification of intracellular cholesterol levels using the Amplex Red Cholesterol Assay Kit confirmed that MβCD significantly reduced cholesterol content in a dose-dependent manner (Fig. 4B). Subsequently, WT and PEX19 KO cells were treated with different concentrations of MβCD (0 mM, 5 mM, 10 mM, and 15 mM) at 37°C for 1 h before being infected with PDCoV (MOI = 1). Samples were collected at 12 h post-infection, and viral replication was assessed by RT-qPCR, TCID_50_ assays, and Western blot analysis. The results showed that MβCD significantly inhibited PDCoV replication in a dose-dependent manner (Fig. 4C through E), indicating that cholesterol reduction can effectively suppress PDCoV replication. We also designed an experiment to evaluate whether exogenous addition of water-soluble cholesterol to WT and PEX19 KO cells can restore PDCoV replication. To this end, PEX19 KO cells were treated with 10 mM MβCD for 1 h, and then water-soluble cholesterol was added to a final concentration of 400 μg/mL and incubated at 37°C for 1 h to restore cellular cholesterol levels, followed by PDCoV infection (MOI = 1). Samples were collected at 12 h post-infection for RT-qPCR, TCID_50_, and Western blot analysis. The results showed that exogenous addition of water-soluble cholesterol significantly restored PDCoV replication (Fig. 4F through H). These consistent results demonstrate that cholesterol is an essential component for PDCoV replication and suggest that PEX19 may inhibit viral replication by downregulating cholesterol levels through its farnesylation modification.

### Farnesylated PEX19 degrades the PDCoV nsp2

**Fig 5:**
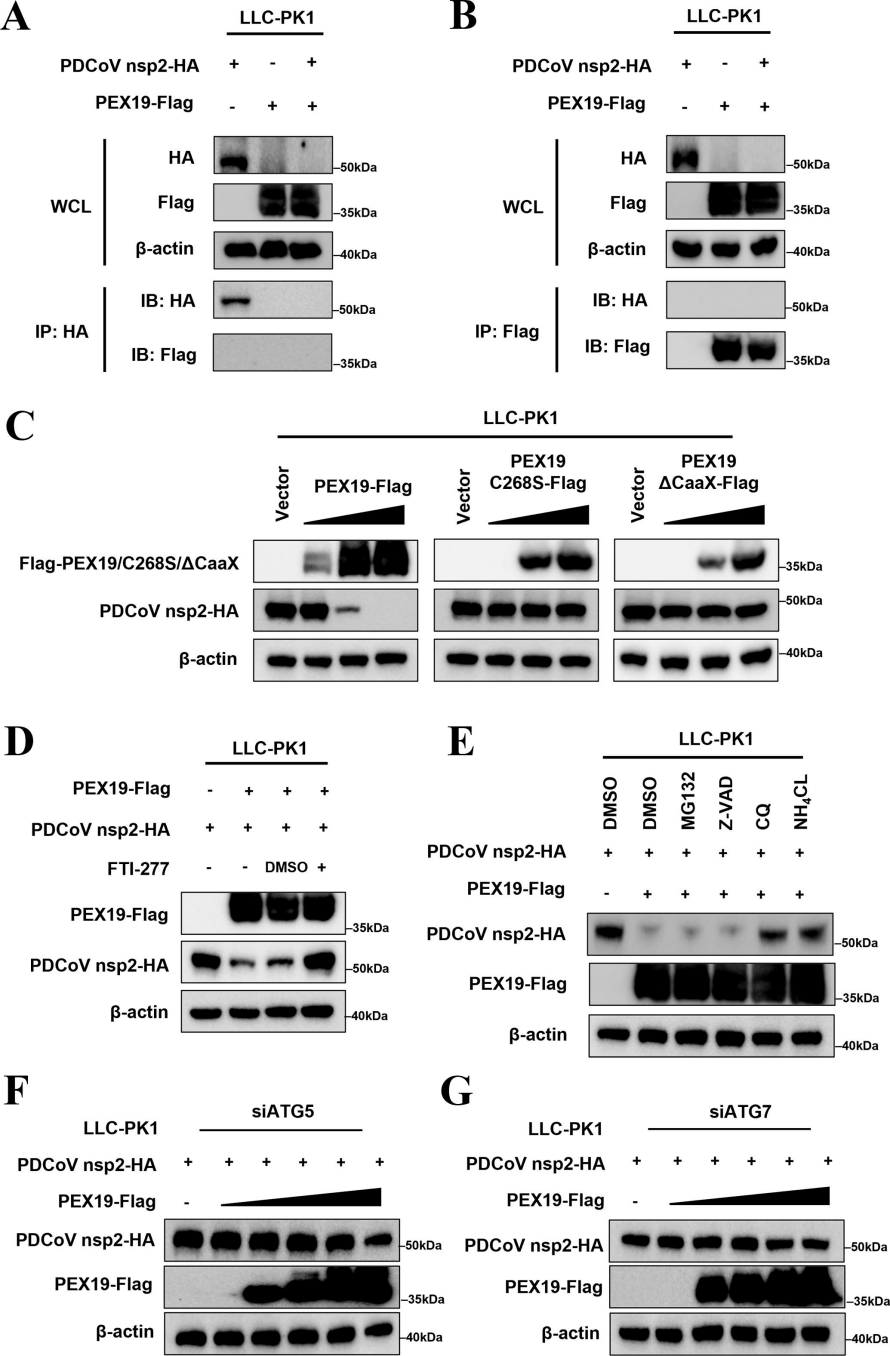
PEX19 promotes the degradation of PDCoV nsp2. (**A and B**) PEX19 reduces the protein levels of PDCoV nsp2. LLC-PK1 cells were co-transfected with pCAGGS-Flag-PEX19 and pCAGGS-HA-nsp2. At 24 h post-transfection, cells were lysed and supernatants collected. A small portion of each lysate was reserved as whole-cell lysate (WCL), and the remaining lysates were subjected to coimmunoprecipitation (Co-IP) using anti-HA (**A**) or anti-Flag (**B**) monoclonal antibodies. (**C**) Farnesylation of PEX19 is required for degradation of PDCoV nsp2. LLC-PK1 cells cultured in 6-well plates were co-transfected with 1 μg of pCAGGS-HA-nsp2 and increasing amounts (0, 0.25, 0.5, and 1 μg) of WT PEX19, PEX19 C268S, or PEX19 ΔCaaX expression constructs. Western blotting was performed using anti-HA, anti-Flag, and anti-β-actin antibodies, respectively. (**D**) FTI-277 abrogates PEX19-mediated degradation of PDCoV nsp2. LLC-PK1 cells were co-transfected with plasmids encoding HA-PDCoV nsp2 and Flag-PEX19. At 18 h post-transfection, cells were treated with 20 μM FTI-277 or DMSO for 6 h. Cells were then harvested for Western blot analysis using anti-HA, anti-Flag, and anti-β-actin antibodies. (**E**) Analysis of the PEX19-mediated degradation pathway of PDCoV nsp2. LLC-PK1 cells were co-transfected with pCAGGS-HA-nsp2 and pCAGGS-Flag-PEX19 or an empty vector. At 12 h post-transfection, cells were treated with DMSO, MG132 (10 μM), Z-VAD (20 μM), chloroquine (CQ, 5 μM), or NH₄Cl (10 mM) for 12 h. Cell lysates were analyzed by Western blotting. (**F and G**) PEX19-mediated degradation of PDCoV nsp2 is impaired in autophagy-deficient cells. LLC-PK1 cells were transfected with siRNA specific to ATG5 (**F**) or ATG7 (**G**). After 24 h, the cells were subsequently co-transfected with pCAGGS-HA-nsp2 and increasing amounts of pCAGGS-Flag-PEX19. Following an additional 24 h, the cells were harvested, and the lysates were analyzed by Western blot using anti-HA and anti-Flag antibodies to detect nsp2 and PEX19, respectively. β-actin was used as a loading control.

To investigate whether PEX19 can directly target viral proteins, we initially performed co-immunoprecipitation (Co-IP) experiments to analyze the interaction between PEX19 and PDCoV-encoded proteins in HEK-293T cells. The results showed that PEX19 could interact with the non-structural protein nsp8 and the accessory protein NS7a proteins, but no degradation of nsp8 or NS7a by PEX19 was observed (Fig. S3). Notably, although no direct interaction was detected between PEX19 and either PDCoV nsp2 or nsp5, whole-cell lysate (WCL) analysis revealed that PEX19 reduced the protein expression levels of both nsp2 (Fig. S4A and B) and nsp5 (Fig. S3), with a more pronounced effect observed on nsp2. Therefore, we selected nsp2 for further investigation and confirmed the PEX19-induced downregulation of nsp2 in LLC-PK1 cells (Fig. 5A and B). Together, these findings suggest that PEX19 may mediate nsp2 degradation through an indirect mechanism.

To elucidate the molecular mechanism underlying nsp2 degradation, we co-transfected LLC-PK1 and HEK-293T cells with PDCoV nsp2 and increasing doses of WT PEX19, PEX19 C268S, or PEX19 ΔCaaX plasmids. Western blot analysis revealed that WT PEX19 markedly reduced nsp2 protein levels in a dose-dependent manner, whereas the farnesylation-deficient mutants (PEX19 C268S and PEX19 ΔCaaX) completely lost their degradation ability (Fig. 5C; Fig. S4C). Consistently, we found that the FTase inhibitor FTI-277 effectively blocked PEX19-mediated nsp2 degradation (Fig. 5D). These results indicate that the degradation of nsp2 by PEX19 is strictly dependent on its farnesylation status.

Cellular protein degradation primarily occurs through three major pathways: apoptosis, autophagy, and the ubiquitin-proteasome system. To determine the specific pathway by which PEX19 degrades PDCoV nsp2, LLC-PK1 and HEK-293T cells were co-transfected with PDCoV nsp2 and PEX19, followed by treatment with DMSO, the proteasome inhibitor MG132, the apoptosis inhibitor Z-VAD-FMK, or the autophagy inhibitors chloroquine (CQ) and NH₄Cl, respectively. Western blot analysis revealed that only CQ and NH₄Cl effectively rescued PEX19-mediated nsp2 degradation (Fig. 5E; Fig. S4D), indicating that PEX19-mediated nsp2 degradation occurs through an autophagy-dependent pathway. To further substantiate this finding, we knocked down the essential autophagy genes, ATG5 and ATG7, in LLC-PK1 cells using siRNA. The results showed that depletion of either ATG5 or ATG7 significantly impaired PEX19-mediated degradation of PDCoV nsp2 (Fig. 5F and G). This finding was further corroborated in HEK-293T cells with knockout (KO) of ATG5 or ATG7, where PEX19 similarly failed to degrade nsp2 (Fig. S4E and F). Collectively, these results demonstrate that farnesylated PEX19 facilitates the degradation of PDCoV nsp2 via the autophagy-lysosome pathway, consequently inhibiting viral replication.

### PEX19 induces low-level interferon production independently of its farnesylation

**Fig 6:**
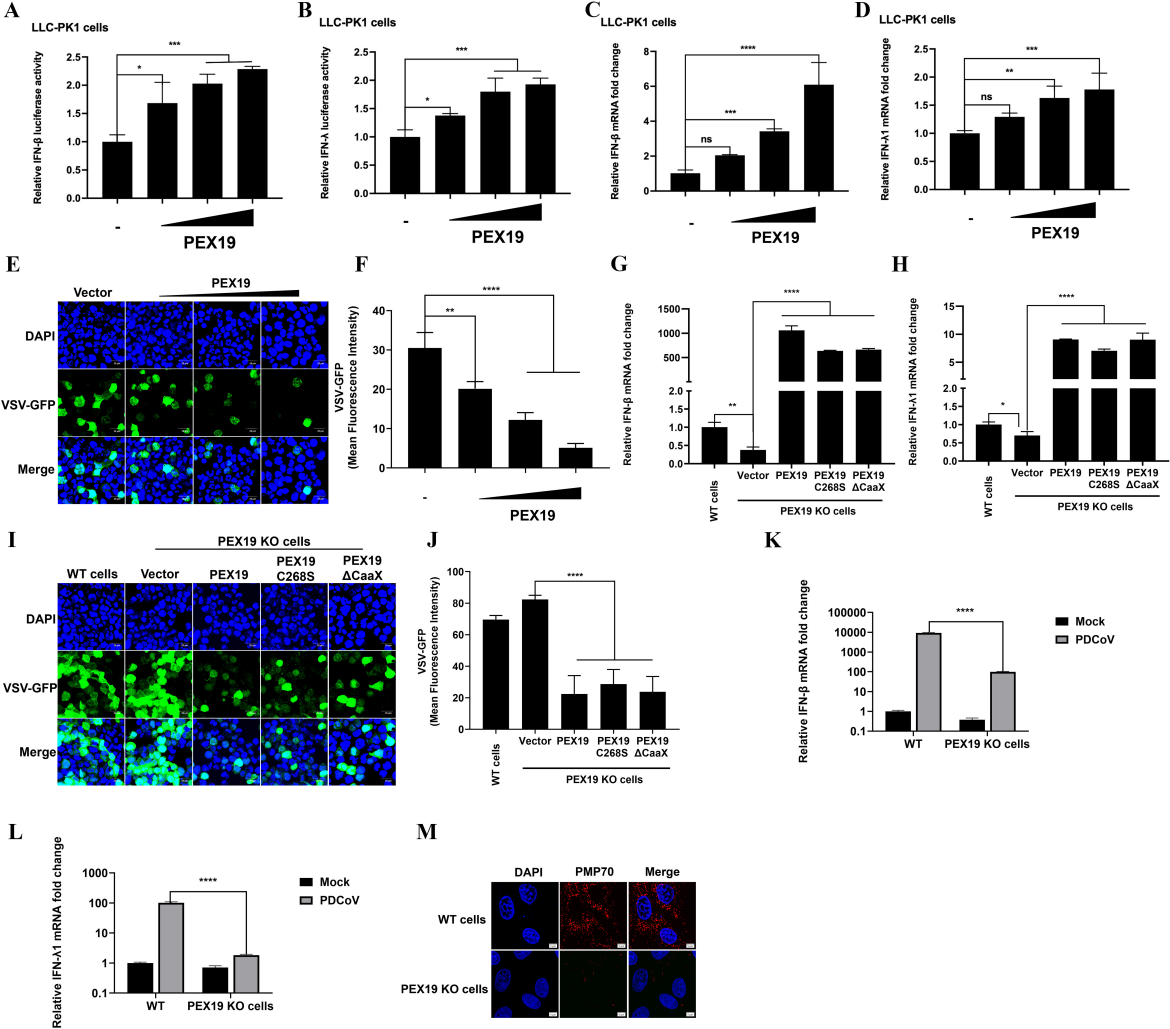
PEX19 induces low-level IFN responses independent of its farnesylation. (**A and B**) Activation of IFN promoters by PEX19. LLC-PK1 cells were co-transfected with IFN-β-Luc or IFN-λ1-Luc reporter plasmids and pRL-TK together with increasing amounts of pCAGGS-Flag-PEX19. Luciferase activity was measured at 24 h post-transfection using a dual-luciferase reporter assay system. (**C and D**) Endogenous IFN induction by PEX19. LLC-PK1 cells were transfected with increasing amounts of pCAGGS-Flag-PEX19, and mRNA levels of IFN-β (**C**) and IFN-λ1 (**D**) were determined by RT-qPCR at 24 h post-transfection. (**E and F**) Induction of IFN by PEX19 assessed by VSV-GFP bioassay. LLC-PK1 cells were transfected with increasing amounts of pCAGGS-Flag-PEX19. At 24 h post-transfection, cell culture supernatants were collected. HEK-293T cells were infected with VSV-GFP (MOI = 0.01) for 1 h, followed by treatment with the collected supernatants for 8 h. (**E**) Viral replication was visualized by fluorescence microscopy. Representative images show VSV-GFP (green) and cell nuclei stained with DAPI (blue). Scale bar, 20 μm. (**F**) Quantification of the mean fluorescence intensity (MFI) of VSV-GFP corresponding to (**E**) using Image *J* software. (**G and H**) Farnesylation-independent induction of IFN by PEX19. PEX19 KO cells were transfected with empty vector, WT PEX19, PEX19 C268S, or PEX19 ΔCaaX constructs. LLC-PK1 WT cells were used as a control. The mRNA levels of IFN-β (**G**) and IFN-λ1 (**H**) were measured by RT-qPCR. (**I and J**) Farnesylation-independent induction of IFN by PEX19. PEX19 KO LLC-PK1 cells were transfected with the indicated constructs. Culture supernatants were harvested at 24 h post-transfection. HEK-293T cells were infected with VSV-GFP (MOI = 0.01) for 1 h and subsequently treated with the supernatants for 12 h. (**I**) Representative images show VSV-GFP (green) and cell nuclei stained with DAPI (blue). Scale bar, 20 μm. (**J**) Quantification of VSV-GFP fluorescence intensity corresponding to (**I**) using Image *J* software. (**K and L**) IFN response to PDCoV infection in LLC-PK1 WT cells and PEX19 KO cells. LLC-PK1 WT cells or PEX19 KO cells were infected with PDCoV (MOI=1). Total RNA was extracted at 12 h post-infection, and IFN-β (**K**) and IFN-λ1 (**L**) mRNA levels were analyzed by RT-qPCR. (**M**) Peroxisome morphology in LLC-PK1 WT cells and PEX19 KO cells. Cells were fixed, and IFA was performed with antibodies against the peroxisomal membrane protein PMP70 (red), and cell nuclei were counterstained with DAPI (blue). Fluorescence was visualized by using a confocal laser scanning microscope. Scale bar, 5 μm.Values are shown as means ± SD. Statistical analyses were performed with one-way ANOVA (for panels A to D, F to H, and J) or two-way ANOVA (for panels K and L) . ns., no significance; *, *P* < 0.05; **, *P* < 0.01; ***, *P* < 0.001; ****, *P* < 0.0001.

Peroxisomes are important platforms for innate immunity. Compared to mitochondria, peroxisomes can activate a transient and rapid type III IFN response. To investigate whether PEX19 can activate IFN production, LLC-PK1 cells were transfected with different doses of pCAGGS-Flag-PEX19. The promoter activity and mRNA levels of IFN-β (type I IFN) and IFN-λ1 (type III IFN) were detected using a dual-luciferase reporter assay system and RT-qPCR. The results showed that PEX19 can dose-dependently activate the promoter activity and mRNA levels of both IFN-β (Fig. 6A and C) and IFN-λ1 (Fig. 6B and D). A similar upregulation of IFN-β and IFN-λ1 mRNA levels was also observed in IPI-2I cells following PEX19 overexpression (Fig. S5A and B). To further determine whether PEX19 can enhance IFN protein levels, interferon bioassays were conducted using vesicular stomatitis virus expressing green fluorescent protein (VSV-GFP), an IFN-sensitive virus. The results showed that supernatants collected from PEX19-overexpressing cells significantly inhibited VSV-GFP replication in a dose-dependent manner compared to those from vector-transfected cells (Fig. 6E and F), indicating elevated IFN protein levels in the culture supernatants.

To further investigate whether PEX19-mediated IFN activation is associated with its farnesylation modification, WT PEX19, PEX19 C268S, and PEX19 ΔCaaX were overexpressed in PEX19 KO LLC-PK1 cells. As illustrated in Fig. 6G and I, RT-qPCR results showed that the baseline mRNA levels of IFN in PEX19 KO cells were significantly lower than those in LLC-PK1 WT cells. Moreover, in PEX19 KO cells, WT PEX19, PEX19 C268S, and PEX19 ΔCaaX significantly upregulated both the mRNA and protein levels of IFN-β and IFN-λ1 (Fig. 6G through J). Consistent with these findings, overexpression of both WT and farnesylation-deficient PEX19 mutants enhanced IFN-β and IFN-λ1 expression in IPI-2I cells (Fig. S5C and D). Furthermore, treatment with FTI-277 did not significantly affect PEX19-induced IFN-β and IFN-λ1 expression in IPI-2I cells (Fig. S5E and F). These results collectively demonstrate that PEX19 activates IFN production independently of its farnesylation modification.

To further verify whether PEX19 promotes IFN production independently of its farnesylation modification during PDCoV infection, we conducted complementation experiments in PEX19 KO cells. The cells were transfected with an empty vector, WT PEX19, PEX19 C268S, or PEX19 ΔCaaX mutants, followed by PDCoV infection. RT-qPCR analysis demonstrated that both WT PEX19 and farnesylation-deficient PEX19 mutants effectively restored IFN-β and IFN-λ1 production to comparable levels (Fig. S5G and H), with no significant differences observed between the WT and mutant groups. Collectively, these results indicate that PEX19 promotes the IFN response through a farnesylation-independent mechanism during PDCoV infection.

The prior results have confirmed that PEX19 knockout enhances PDCoV replication (Fig. 1G and H). To determine whether this effect is associated with impaired production of IFN, we compared the IFN response following PDCoV infection in LLC-PK1 WT cells and PEX19 KO cells. The results showed that, compared to LLC-PK1 WT cells, PEX19 KO cells exhibited a significantly attenuated IFN-β response (Fig. 6K), while IFN-λ1 production was nearly abolished (Fig. 6L). This phenomenon is consistent with the preferential activation of type III IFN by peroxisomes (41, 42). Additionally, peroxisomes were labeled using the peroxisomal membrane protein PMP70, and IFA analysis revealed an obvious reduction in peroxisomal numbers in PEX19 KO cells, with peroxisomes almost absent compared to WT cells (Fig. 6M). These findings suggest that PEX19 may play a crucial role in maintaining peroxisomal structural integrity, which is essential for proper activation of the IFN signaling pathway, thereby inhibiting PDCoV replication.

## DISCUSSION

Peroxisomes have been demonstrated to play important roles during various viral infections. On one hand, peroxisome-mediated lipid synthesis is essential for the infection of many enveloped viruses (26, 43, 44); on the other hand, peroxisomes can serve as platforms for innate immune responses to restrict viral replication (45–47). This dual role positions peroxisomes at the center of an intriguing interplay between viruses and host cells. Understanding how peroxisomes regulate PDCoV infection is critical for identifying new antiviral targets. In this study, we uncovered three kinds of mechanisms by which PEX19, dependent or independent of its farnesylation, inhibits PDCoV replication, thereby expanding our understanding of host antiviral defenses.

Here, we identified PEX19 as a host restriction factor against PDCoV infection. Although the detection of farnesylated PEX19 remains technically challenging due to the lack of commercially available antibodies for protein farnesylation detection, our findings demonstrate that the antiviral activity of PEX19 is largely associated with its farnesylation, as evidenced by site-directed mutagenesis (C268S and ΔCaaX) and pharmacological inhibition (FTI-277). However, the functional significance of PEX19 farnesylation has been a subject of debate. Some studies suggest that PEX19 farnesylation is required for its structural integrity and peroxisome biogenesis (25, 48, 49), while others indicate that farnesylation is dispensable for peroxisome formation (50–52). In eukaryotic cells, the mevalonate pathway converts acetyl-CoA into FPP, which serves both as a precursor for cholesterol synthesis and as a substrate for protein farnesylation (53). Previous studies have shown that cholesterol accumulates in cells and mouse models with peroxisomal biogenesis disorders (38, 54), suggesting that peroxisomes contribute to cholesterol metabolism. Thus, it is plausible that PEX19 regulates cellular cholesterol levels either by competing for FPP to support its own farnesylation, thereby reducing cholesterol biosynthesis, or by directly affecting peroxisomal function via farnesylation, subsequently influencing cholesterol metabolism. The results of our present study provide evidence supporting this hypothesis. Using the Amplex Red Cholesterol Assay Kit and the cholesterol-specific dye Filipin, we demonstrated that overexpression of PEX19 reduces cellular cholesterol levels. Notably, farnesylation-deficient PEX19 mutants display a markedly diminished capacity to lower cellular cholesterol levels, suggesting that PEX19 may modulate cholesterol biosynthesis by competing for the intracellular FPP pool. Furthermore, considering that PDCoV infection significantly downregulates PEX19 expression, we propose that PDCoV-induced downregulation of PEX19 attenuates its inhibitory effect on cholesterol biosynthesis, thereby creating a membrane environment favorable for PDCoV replication and assembly.

Cholesterol is a critical component of lipid rafts and plays important roles throughout various stages of the viral life cycle (55), including viral entry, which, for many enveloped viruses, depends on the presence of cholesterol in both viral and cellular membranes (56). It is well established that cholesterol is essential for the replication of various coronaviruses, including SARS-CoV, type I FCoV, TGEV, and PDCoV (56–60). Consistently, we confirmed that the depletion of cholesterol by MβCD inhibited PDCoV replication, while supplementation with exogenous cholesterol rescued viral growth. These findings suggest that PEX19 may suppress PDCoV replication through its farnesylation-dependent modulation of cellular cholesterol levels.

Many studies have revealed that PEX19 can interact with various viral proteins. For instance, the vMIA protein of human cytomegalovirus (HCMV) interacts with PEX19 and is translocated to the peroxisomal membrane, where it inhibits the oligomerization of mitochondrial antiviral signaling protein (MAVS), blocks downstream antiviral signaling, and facilitates immune evasion (45). Similarly, the vFLIP protein of Kaposi’s sarcoma-associated herpesvirus (KSHV) relies on PEX19 for peroxisomal localization, where it interacts with peroxisomal MAVS to establish latent infection (61). In addition, the capsid proteins of several flaviviruses have been shown to bind PEX19, resulting in a significant reduction of PEX19 and other peroxisomal proteins in infected cells (28, 62). More recently, the M2 protein of influenza A virus (IAV) was also found to interact with PEX19, disrupting peroxisomal function to promote viral replication (63). However, whether PEX19 interacts with coronavirus-encoded proteins has not yet been explored. In this study, we performed Co-IP assays and identified PDCoV nsp8 and NS7a proteins as PEX19-interacting partners; however, PEX19 does not degrade nsp8 or NS7a. The underlying biological significance of these interactions remains unclear and will be a focus of future investigations.

To our surprise, PEX19 markedly reduced the protein levels of several PDCoV proteins, such as nsp2, nsp5, and the S protein, in whole-cell lysates, although no direct interaction band between PEX19 and these viral proteins was detected. Notably, the reduction in nsp2 was the most pronounced; therefore, we prioritized nsp2 for further investigation to elucidate the potential mechanism underlying the antiviral activity of PEX19. Coronavirus nsp2 is an N-terminal cleavage product of the viral polyprotein and exhibits considerable sequence diversity. Previous studies have demonstrated that nsp2 plays important roles during viral infection. For example, SARS-CoV-2 nsp2 has been predicted to be critical for the initiation of viral transcription and translation (64). Additionally, PEDV nsp2 functions as a novel virulence factor that suppresses innate antiviral immunity by targeting TBK1 to induce NBR1-mediated selective autophagy (65), while TGEV nsp2 is implicated in the regulation of inflammatory responses (66). Recent evidence further indicates that PDCoV nsp2 targets STING, thereby suppressing cGAS-STING-mediated production of type I and III IFN (67). Thus, the degradation of nsp2 by PEX19 may contribute, at least in part, to the induction of IFN production observed upon PEX19 overexpression. However, the mechanism by which PEX19 promotes nsp2 degradation remains unclear. Given that no direct interaction between PEX19 and nsp2 was detected, we postulate that PEX19 facilitates nsp2 degradation through an indirect mechanism rather than direct binding. This process may involve a host autophagy cargo receptor or adapter protein that serves as a molecular bridge between PEX19 and nsp2.

Furthermore, we demonstrated that PEX19 promotes the degradation of PDCoV nsp2 through the autophagy-lysosome pathway. However, the precise molecular mechanisms by which PEX19 induces autophagy remain unclear. A previous study has suggested that PEX19 is required for general autophagy (68). Additionally, other research has shown that PEX19 can bind to the transmembrane domain of MARCH5, facilitating its targeting to peroxisomes and thereby mediating pexophagy (69). Notably, our data indicate that only farnesylated PEX19 effectively promotes the degradation of PDCoV nsp2, suggesting a critical role for farnesylation in PEX19-mediated autophagy.

Peroxisomes have been recognized as critical platforms for early innate immune responses and play essential roles in antiviral immunity (41, 70). Emerging studies have highlighted the importance of PEX19 in peroxisome-mediated innate immunity. For instance, PEX19 interacts with the IFN-stimulated gene product Viperin to enhance antiviral innate immune responses (71). Several members of the Flaviviridae family, including West Nile virus, dengue virus, and Zika virus, have been shown to antagonize IFN signaling by binding to PEX19 through their capsid proteins, thereby impairing peroxisome biogenesis (28, 47, 72). Recent evidence further suggests that although PEX19 deficiency does not affect type I IFN transcription upon IAV infection, it significantly reduces type III IFN production, indicating that PEX19 plays a critical role in peroxisomal MAVS-mediated type III IFN responses (63).

In this study, we found that PEX19 could dose-dependently activate both type I and type III IFN production; however, the magnitude of activation was relatively modest, suggesting that under physiological conditions, PEX19-mediated innate immune activation is maintained at a low level. In PEX19 KO cells, reintroduction of PEX19 robustly induced IFN production independently of its farnesylation modification. Moreover, the induction of IFN-β by PDCoV infection was markedly diminished in PEX19 KO cells, and the production of IFN-λ1 was almost completely abolished. These observations are consistent with findings from Liu in the context of IAV infection (63) and align with the known preference of peroxisomes for promoting type III IFN responses. Despite these findings, the mechanisms by which PEX19 stimulates IFN signaling remain largely unclear. Here, we demonstrate that PEX19-induced IFN production is dependent on the MAVS-mediated RLR signaling pathway, as MAVS knockdown significantly attenuated PEX19-induced upregulation of both IFN-β and IFN-λ1 (Fig. S6).

It is worth noting that our initial screening revealed that, in addition to PEX19, several other peroxins also displayed varying degrees of inhibitory effects against PDCoV infection (Fig. 1). However, the mechanisms by which these peroxins, apart from PEX19, restrict PDCoV replication remain uninvestigated. Notably, PEX13 has been reported to promote immune responses by regulating peroxisome abundance and mitochondrial antiviral signaling protein (MAVS)-mediated IFN-III signaling (73); PEX7 participates in peroxisomal biogenesis, which supports innate immune activation (74); and PEX11β regulates peroxisome proliferation, thereby enhancing antiviral responses (75). Therefore, these peroxins may inhibit PDCoV infection through pathways distinct from the PEX19-mediated cholesterol reduction and nsp2 degradation characterized in this study, and the specific mechanisms underlying their antiviral activities warrant further investigation.

**Fig 7:**
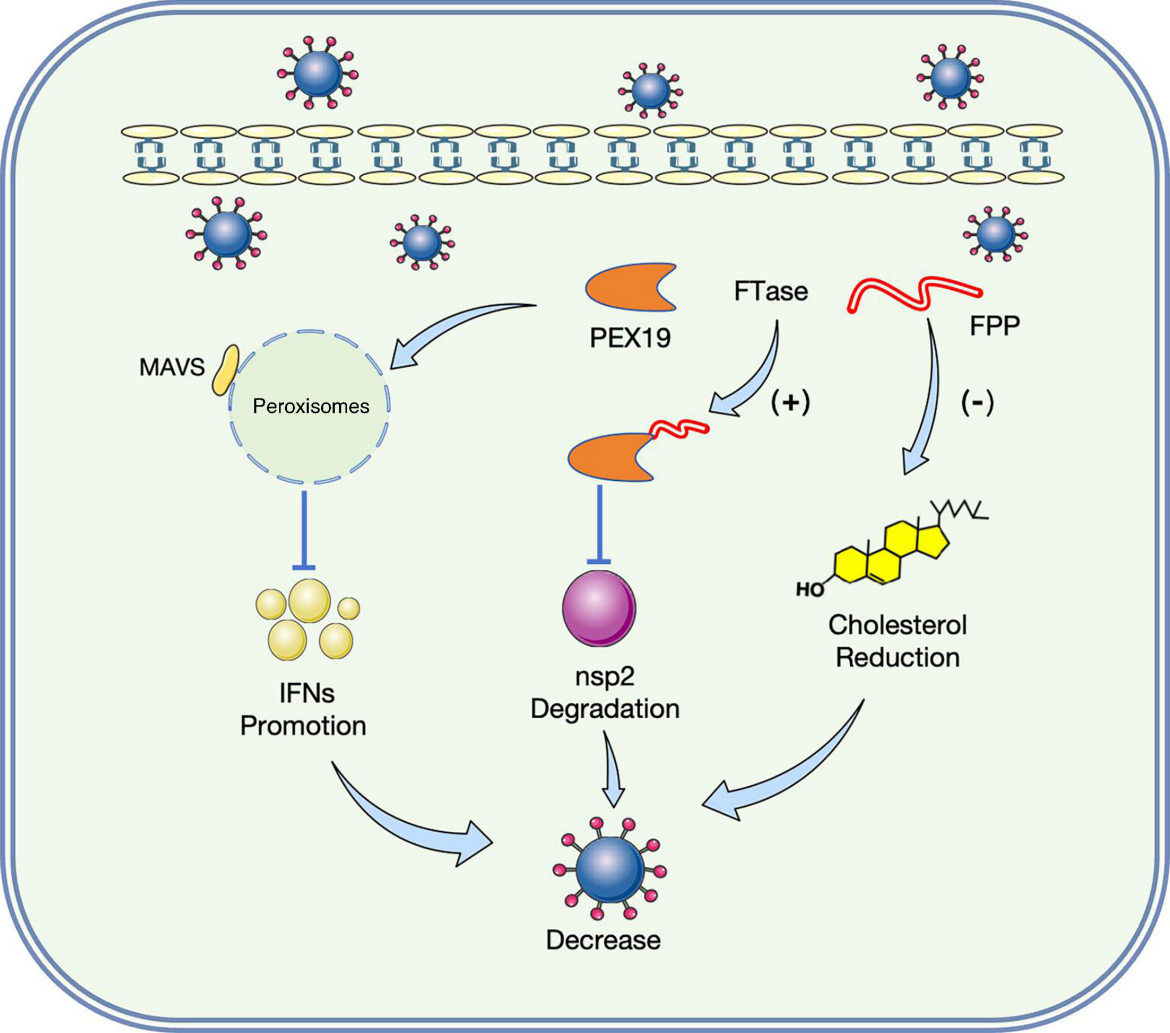
Proposed model of PEX19-mediated restriction of PDCoV replication. PEX19 functions as a host restriction factor that antagonizes PDCoV infection primarily in a farnesylation-dependent manner. Three mechanisms were identified: (i) farnesylated PEX19 reduces cellular cholesterol levels, thereby impairing PDCoV replication; (ii) farnesylated PEX19 specifically targets PDCoV nonstructural protein nsp2 for degradation, limiting viral replication; and (iii) PEX19 induces low-level IFN responses in a farnesylation-independent manner.

To further elucidate the interplay between PEX19 and PDCoV, we investigated the effect of PDCoV infection on PEX19 protein expression. Our findings demonstrate that PDCoV infection significantly reduces PEX19 expression in both LLC-PK1 cells and the intestinal tissues of infected piglets (Fig. S7). Given the potential for PDCoV to infect humans, the development of effective antiviral agents is imperative. Considering the established antiviral activity of PEX19 and its downregulation during PDCoV infection, we propose that targeting PEX19 represents a promising antiviral strategy. Specifically, the development of compounds that stabilize PEX19 expression or enhance its farnesylation may offer a potential approach for controlling PDCoV infection. In summary, our study provides the first evidence that PEX19 exhibits antiviral activity against PDCoV and establishes that its antiviral function is closely associated with its farnesylation modification. We identified three distinct mechanisms by which PEX19 inhibits PDCoV replication: suppression of cholesterol biosynthesis, selective degradation of PDCoV nsp2, and activation of IFN-mediated innate immune responses (Fig. 7). These findings not only advance our understanding of the interplay between PDCoV and the host but also suggest potential strategies for antiviral immune therapies targeting PEX19.

## MATERIALS AND METHODS

### Cells, virus, and reagents

Porcine intestinal epithelial cells (IPI-2I) and human embryonic kidney (HEK-293T) cells were obtained from the China Center for Type Culture Collection (Wuhan, China). LLC porcine kidney (LLC-PK1) cells were purchased from the American Type Culture Collection (ATCC CL-101; Manassas, VA, USA). LLC-PK1 cells were cultured in modified Eagle’s medium (MEM; Hyclone, USA). IPI-2I and HEK-293T cells were cultured in Dulbecco’s modified Eagle’s medium (DMEM; Gibco, USA) supplemented with 10% fetal bovine serum (FBS; TransGen, China). PDCoV strain CHN-HN-2014 (GenBank accession number KT336560) used in this study was isolated from a piglet with severe diarrhea in China in 2014 (76). Antibodies directed against PEX19 and β-actin were purchased from ABclonal (China). Mouse and rabbit anti-DDDDK (Flag) and anti-hemagglutinin (HA) mAbs were purchased from Medical and Biological Laboratories (MBL, Japan). Mouse anti-PDCoV N protein mAb was prepared in our laboratory, as described previously (77). Inhibitors MG132 and Z-VAD were purchased from Beyotime (Shanghai, China), while FTI-277, chloroquine (CQ), and ammonium chloride (NH_4_Cl) were sourced from MedChemExpress (MCE, Shanghai, China). Cholesterol dye Filipin was purchased from Sigma (Catalog No.: SAE0087), and the Amplex Red Cholesterol Assay Kit was obtained from Thermo Fisher Scientific (Catalog No.: A12216).

### Plasmid construction and siRNA sequences

Full-length cDNAs of PEX5, PEX7, PEX11α, PEX11β, PEX11γ, PEX12, PEX14, PEX16, and PEX19 were amplified from LLC-PK1 cells using specific primers and cloned into the pCAGGS vector with an N-terminal Flag tag to generate pCAGGS-Flag-PEXs. The eukaryotic expression plasmids encoding PDCoV proteins and the luciferase reporter plasmids IFN-β-Luc, IFN-λ1-Luc, and pRL-TK (internal control) were described in our previous studies (78, 79). Farnesylation site mutants of PEX19 were generated by site-directed mutagenesis using pCAGGS-Flag-PEX19 as a template. The cysteine at position 268 was substituted with serine (PEX19 C268S), and the C-terminal four amino acids (CaaX) were deleted (PEX19 ΔCaaX). All constructs were confirmed by DNA sequencing. For siRNA-mediated gene silencing, the following siRNA sequences were used: siATG5 (sense: 5′- GGA UGU AAU UGA AGC UCA UTT-3′, antisense: 5′- AUG AGC UUC AAU UAC AUC CTT-3′), siATG7 (sense: 5′- GCA UCA UCU UCG AAG UGA ATT-3′, antisense: 5′- UUC ACU UCG AAG AUG AUG CTT-3′), and siMAVS (sense: 5′- GAG AGG AUG AGC CAA GUU ATT-3′, antisense: 5′- UAA CUU GGC UCA UCC UCU CTT-3′). For transfection assays, plasmids or siRNA were transfected into cells using JetPRIME (Polyplus, Illkirch, France) according to the manufacturer’s instructions.

### Co-IP assay

Cells were transfected with plasmids for the indicated times and then lysed in lysis buffer. The lysates were incubated on a rotator at 4°C for 30 min. A portion of the supernatant was designated as the WCL fraction. The remaining portions of the supernatants from lysed cells were incubated with the appropriate antibody at 4°C overnight on a rotator and then treated with protein A+G agarose beads for 6 h at 4°C. The beads were then washed three times with lysis buffer, with each wash involving incubation at 4°C for 15 min on a slow rotator, followed by centrifugation at 3,000 r/min for 5 min. Finally, the beads were resuspended in 50 μL of lysis buffer, mixed with 5× SDS PAGE loading buffer, boiled at 100°C for 10 min, and then subjected to Western blot.

### Western blot

Western blot analysis was performed as previously described (80). Briefly, cell lysates were prepared using lysis buffer containing 20 mM Tris-HCl (pH 7.5), 150 mM NaCl, 1% Triton X-100, 1 mM EDTA, and 10% glycerol, along with a protease inhibitor cocktail (Beyotime). Protein samples were resolved by SDS-PAGE and subsequently transferred onto polyvinylidene difluoride membranes (Millipore, USA). The membranes were blocked with non-fat milk and incubated with the indicated primary antibodies, followed by HRP-conjugated secondary antibodies (Beyotime). Protein bands were visualized using enhanced chemiluminescence reagents (Bio-Rad, USA). β-actin was used as the loading control.

### VSV-GFP bioassay for IFN activity

The recombinant vesicular stomatitis virus expressing green fluorescent protein (VSV-GFP) was preserved in our laboratory. To evaluate the secretion of functional IFN protein, HEK-293T cells were seeded in 24-well plates and subsequently infected with VSV-GFP at an MOI of 0.01. After viral adsorption for 1 h at 37°C, the inoculum was removed, and the cells were incubated with the indicated harvested supernatants. Viral replication was monitored using a fluorescence microscope, and the mean fluorescence intensity (MFI) was quantified using ImageJ software.

### Generation of KO cell lines

The PEX19 KO cell lines were generated by the CRISPR/Cas9 system as described previously (81). Briefly, LLC-PK1 cells were transfected with PX459 expressing sgRNAs (sgRNA-F1: 5¢-GGAGCTGTTCGACAGCGAGT-3′, sgRNA-F2: 5¢-AGCCACTGCAGAGTTCGAGA-3¢, sgRNA-F3: 5′-CTGCAGAGTTCGAGAAGGCA-3′), and then selected with puromycin for 36–48 h. The surviving cells were cultured to generate polyclonal cell lines with individual molecular KOs. The genotype of each single-cell clone was identified with Sanger sequencing, and protein expression was verified with a western blot analysis.

### RNA extraction and RT-qPCR

Total cellular RNA was extracted using TRIzol Reagent (Invitrogen, USA) and subsequently reverse transcribed to cDNA with HiScript II Q RT SuperMix for qPCR (Vazyme, China), according to the manufacturer’s protocol. The cDNA was then subjected to qPCR with SYBR qPCR Master Mix (Vazyme, China) with specific primers (for the PDCoV nsp16 gene: 5′-GCTACGGCCTGTAAGCTAAA-3′/5′-CGTCCTGATGCAACGAGATAG-3′; for the PDCoV N gene: 5′-AGCTGCTACCTCTCCGATTC-3′/5′-ACATTGGCACCAGTACGAGA-3′; for the IFN-β gene: 5′-GCTAACAAGTGCATCCTCCAAA-3′/5′-AGCACATCATAGCTCATGGAAAGA-3′; for the IFN-λ1 gene: 5′-AACTTCAGGCTTGCATCAGG-3′/5′-TCTTTCTTTGTGGCTTCTTGG-3′; for the GAPDH gene: 5′-ACATGGCCTCCAAGGAGTAAGA-3′/5′-GATCGAGTTGGGGCTGTGACT-3′).

### Immunofluorescence assay

IFA was conducted following established protocols (82). Briefly, cells cultured on microscope coverslips were fixed with 4% paraformaldehyde, permeabilized with 0.1% Triton X100, and blocked with bovine serum albumin (BSA). Subsequently, the cells were incubated with specific primary antibodies, followed by the corresponding Alexa Fluor 488- or 594-conjugated secondary antibodies. Nuclei were stained with 4′,6-diamidino-2-phenylindole (DAPI; Beyotime) or propidium iodide (PI; Beyotime). Fluorescence images were acquired using a confocal laser scanning microscope (Fluoview v.3.1; Olympus, Japan).

### TCID_50_ assay

Viral titers were measured with a TCID_50_ assay. Briefly, the samples were serially diluted 10-fold from 10^−1^ to 10^−9^ with MEM containing 7.5 μg/mL trypsin. Confluent LLC-PK1 cells in 96-well plates were inoculated (in octuplicate) with each dilution (100 μL/well) and incubated at 37°C for 2–3 days. The cytopathic effects (CPEs) were observed and recorded, and the viral titers (TCID_50_/mL) were calculated with the Reed–Muench method.

### Dual luciferase reporter assay

The promoter activities of IFN-β and IFN-λ1 were analyzed as described previously (83). Briefly, cells were co-transfected with the target reporter plasmids (IFN-β-*Luc* or IFN-λ1-*Luc*) and the internal control plasmid pRL-TK (Promega, USA). At 24 h post-transfection, cell lysates were collected, and both firefly and Renilla luciferase activities were measured by the Dual-Luciferase Reporter Assay System (Promega). The data are presented as relative luciferase activity, which was calculated by normalizing firefly luciferase activity to Renilla luciferase activity.

### Cellular cholesterol content measurement

LLC-PK1 cells were seeded in six-well plates and transfected with 2 µg of WT PEX19, PEX19 C268S, or PEX19 ΔCaaX plasmids. After 24 h of transfection, cells were harvested using 0.25% trypsin-EDTA. Cholesterol levels in collected cells were measured using a commercial cholesterol assay kit (Amplex Red Cholesterol Assay Kit, Thermo Fisher Scientific, USA) according to the manufacturer’s instructions. Additionally, cholesterol levels were analyzed in cells treated with methyl-β-cyclodextrin (MβCD).

### Filipin staining

Cholesterol accumulation was assessed using filipin (Sigma-Aldrich, USA). LLC-PK1 cells transfected with WT PEX19, PEX19 C268S, or PEX19 ΔCaaX plasmids were fixed with 4% paraformaldehyde for 15 min, followed by permeabilization with methanol for 10 min. Cells were then incubated with filipin stain at room temperature for 1 h. After washing with phosphate-buffered saline (PBS), cells were counterstained with propidium iodide (PI; MCE, USA) for 15 min. Fluorescence images were captured using a confocal laser scanning microscope (Olympus Fluoviewer 3.1, Japan).

### Cell viability assay

Cytotoxicity was assessed using the Cell Counting Kit-8 (CCK-8; Beyotime) following the manufacturer’s protocol, as previously reported (82).

### Statistical analysis

All experimental data are given as means ± standard deviations (SD). Statistical differences were evaluated with Student’s *t* test or one-way ANOVA using GraphPad Prism 8 (ns, not significant; *, *P* ≤ 0.05; **, *P* ≤ 0.01; ***, *P* ≤ 0.001; ****, *P* ≤ 0.0001).

## Data Availability

All methods and data described in this article are available from the corresponding author upon request.

## SUPPLEMENTAL MATERIAL

Supplemental figures jvi.02097-25-s0001.docx Fig. S1 to S7.

ASM does not own the copyrights to Supplemental Material that may be linked to, or accessed through, an article. The authors have granted ASM a non-exclusive, world-wide license to publish the Supplemental Material files. Please contact the corresponding author directly for reuse.
