# Supplementary material for: PEX19 restricts porcine deltacoronavirus replication through farnesylation-dependent and -independent mechanisms
Source: J Virol. 2026 Mar 24;100(4):e02097-25. doi: 10.1128/jvi.02097-25 (PMC13098205; doi:10.1128/jvi.02097-25)
Supplement: Supplemental figures — Fig. S1 to S7. [file jvi.02097-25-s0001.docx]

**Supplementary Materials**

**Fig. S1.** Effects of PEX proteins on PDCoV RNA levels and PEX19 knockout on cell viability.

**Fig. S2.** Sequence alignment of PEX19 homologs across different species and the effects of wild-type and mutant PEX19 on PDCoV replication.

**Fig. S3.** Co-IP screening of PDCoV-encoded proteins that interact with PEX19.

**Fig. S4.** PEX19 promotes the degradation of PDCoV nsp2.

**Fig. S5.** PEX19 induces low-level IFN responses.

**Fig. S6.** PEX19 promotes IFN production via MAVS.

**Fig. S7.** Effect of PDCoV infection on the expression of endogenous PEX19 protein.

**Fig. S1**

**
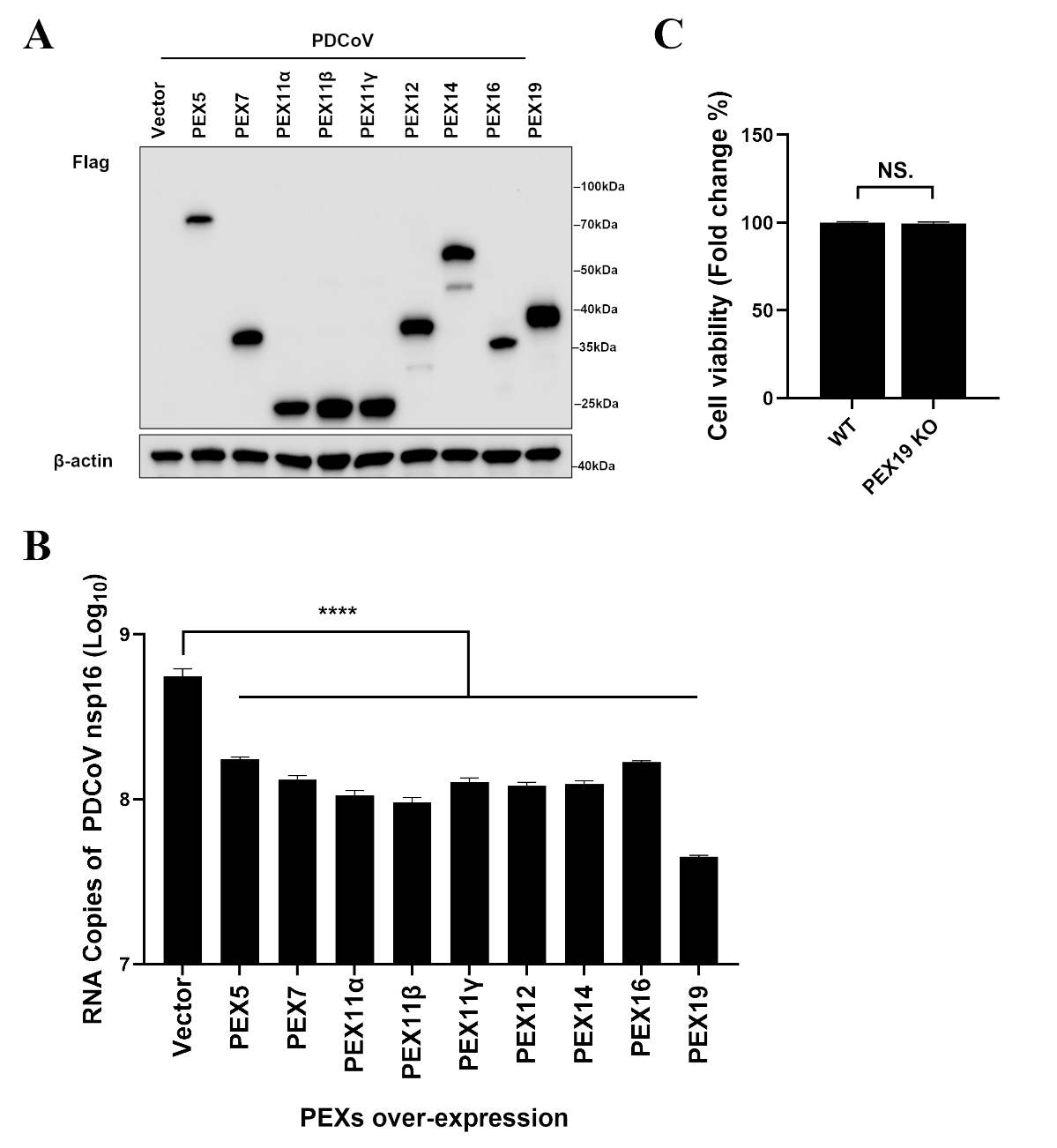
**

**Fig. S1.** **Effects of PEX proteins on PDCoV RNA levels and PEX19 knockout on cell viability. (A–B)** LLC-PK1 cells were transfected with plasmids encoding PEX5, PEX7, PEX11α, PEX11β, PEX11γ, PEX12, PEX14, PEX16, PEX19, or an empty vector as a control. At 12 h post-transfection, cells were infected with PDCoV at a 1 MOI for an additional 12 h. (A) Cell lysates were harvested for western blot analysis to identify the expression of each PEX protein. (B) Total RNA was extracted, and viral nsp16 RNA levels were quantified by RT‑qPCR to evaluate viral replication. **(C)** Cell viability of wild-type (WT) and PEX19 KO LLC-PK1 cells was assessed using the CCK-8 assay.

**Fig. S2**

**
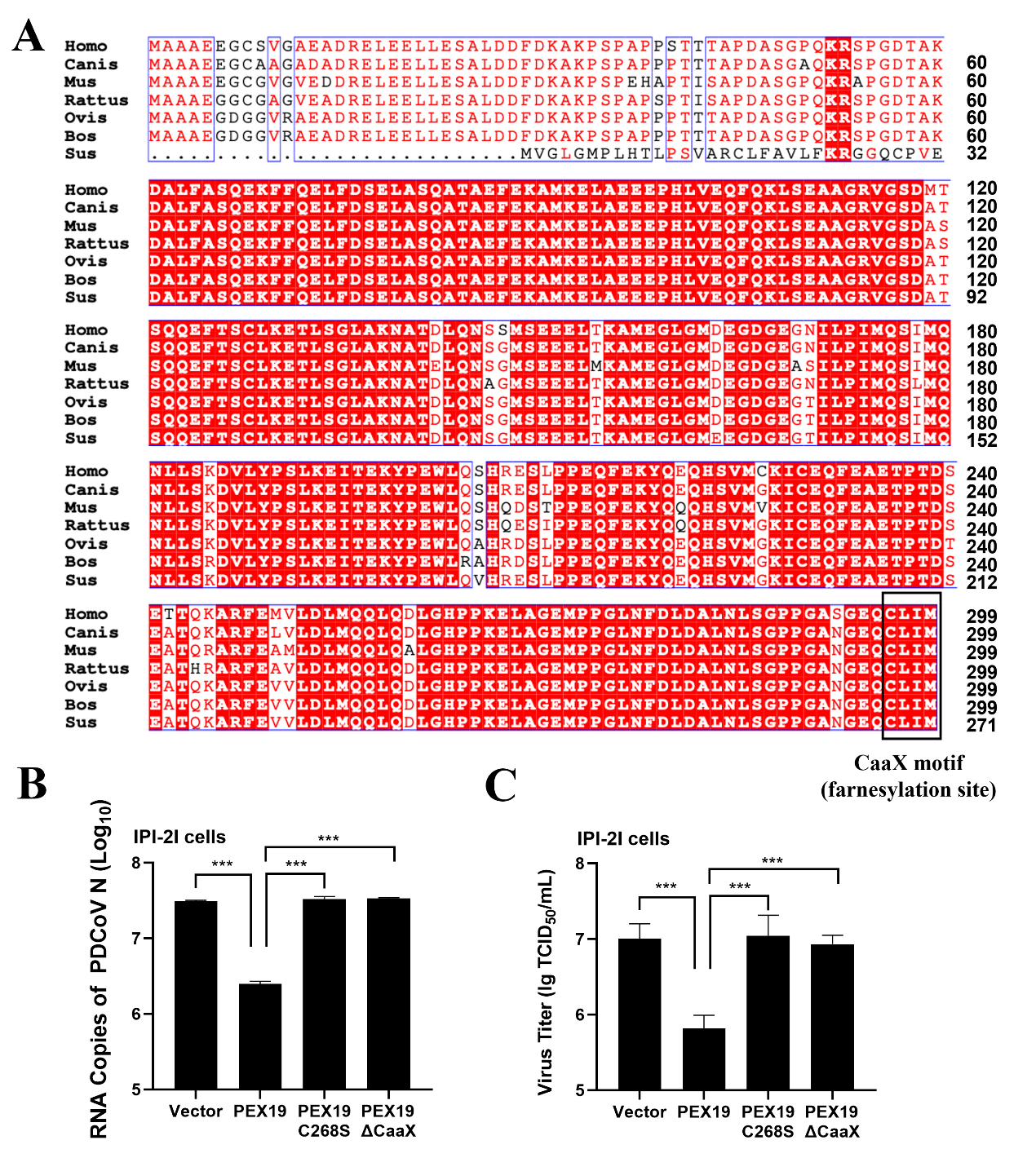
**

**Fig. S2. Sequence alignment of PEX19 homologs across different species and the effects of wild-type and mutant PEX19 on PDCoV replication. (A)** Amino acid sequences of PEX19 from Homo sapiens (GenBank accession number: BAA76291.1), Canis lupus familiaris (GenBank accession number: XP_038442279.1), Mus musculus (GenBank accession number: NP_075528.3), Rattus norvegicus (GenBank accession number: NP_001100845.1), Ovis aries (GenBank accession number: XP_027832857.1), Bos taurus (GenBank accession number: NP_001029712.1), and Sus scrofa (GenBank accession number: XP_013852328.1) were aligned using the CLUSTALW algorithm (https://www.genome.jp/tools-bin/clustalw). Identical residues are highlighted in white, and similar residues are shown in red. The conserved C-terminal CaaX farnesylation motif is indicated. **(B–C)** IPI-2I cells were transfected with plasmids encoding WT PEX19 or its mutant variants (PEX19 C268S and PEX19 ΔCaaX). At 12 h post-transfection, cells were infected with PDCoV (MOI = 0.5). Cells were then collected at 18 hpi for RT-qPCR (B) and TCID_50_ assays (C).

**Fig. S3**

**
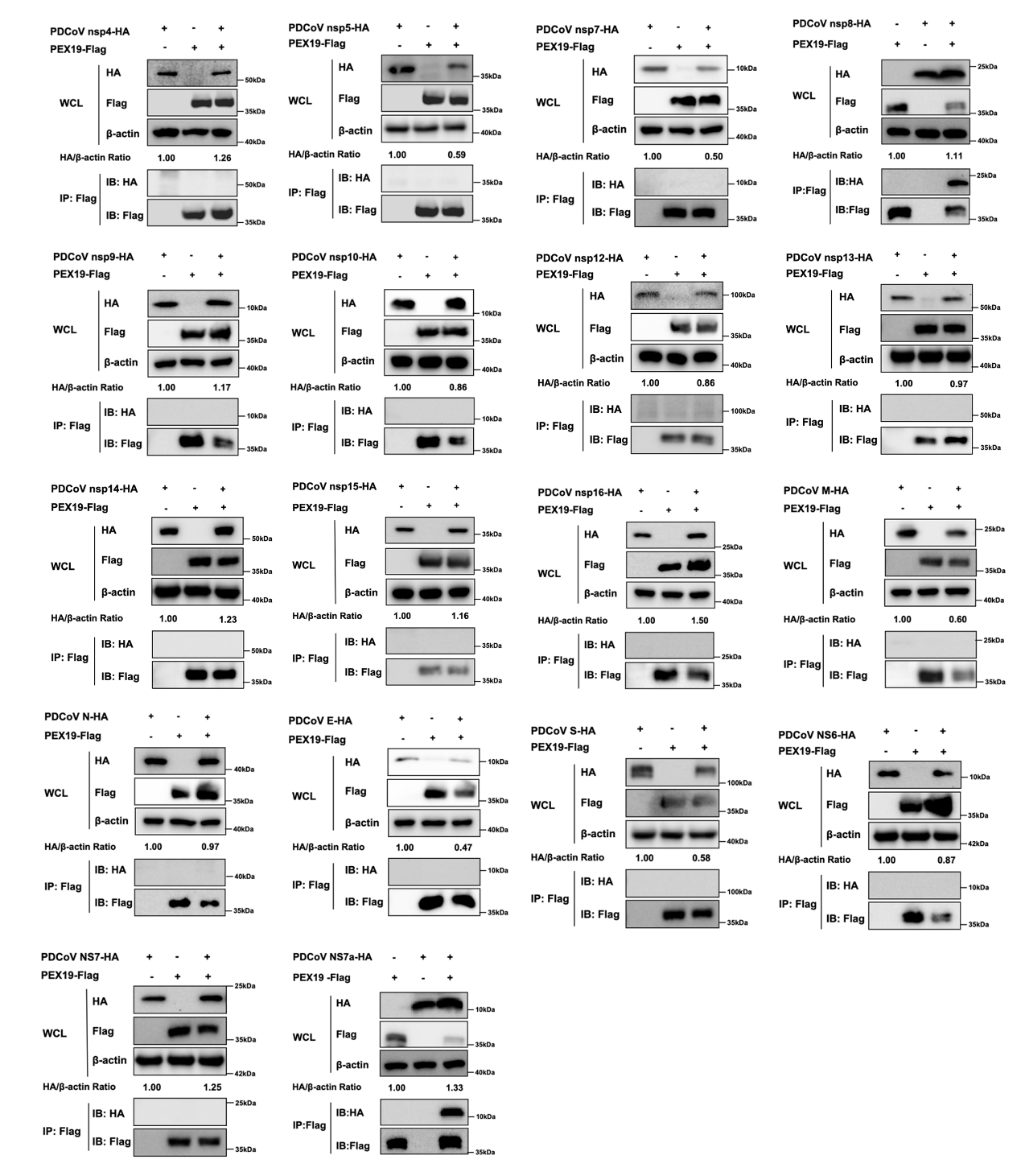
**

**Fig. S3. Co-IP screening of PDCoV-encoded proteins that interact with PEX19.** HEK-293T cells were co-transfected with pCAGGS-Flag-PEX19 and expression constructs of 18 individual PDCoV-encoded proteins, including 11 nonstructural proteins, 4 structural proteins, and 3 accessory proteins. At 24 h post-transfection, cell lysates were subjected to Co-IP using anti-Flag antibody to assess protein–protein interactions.

**Fig. S4**

**
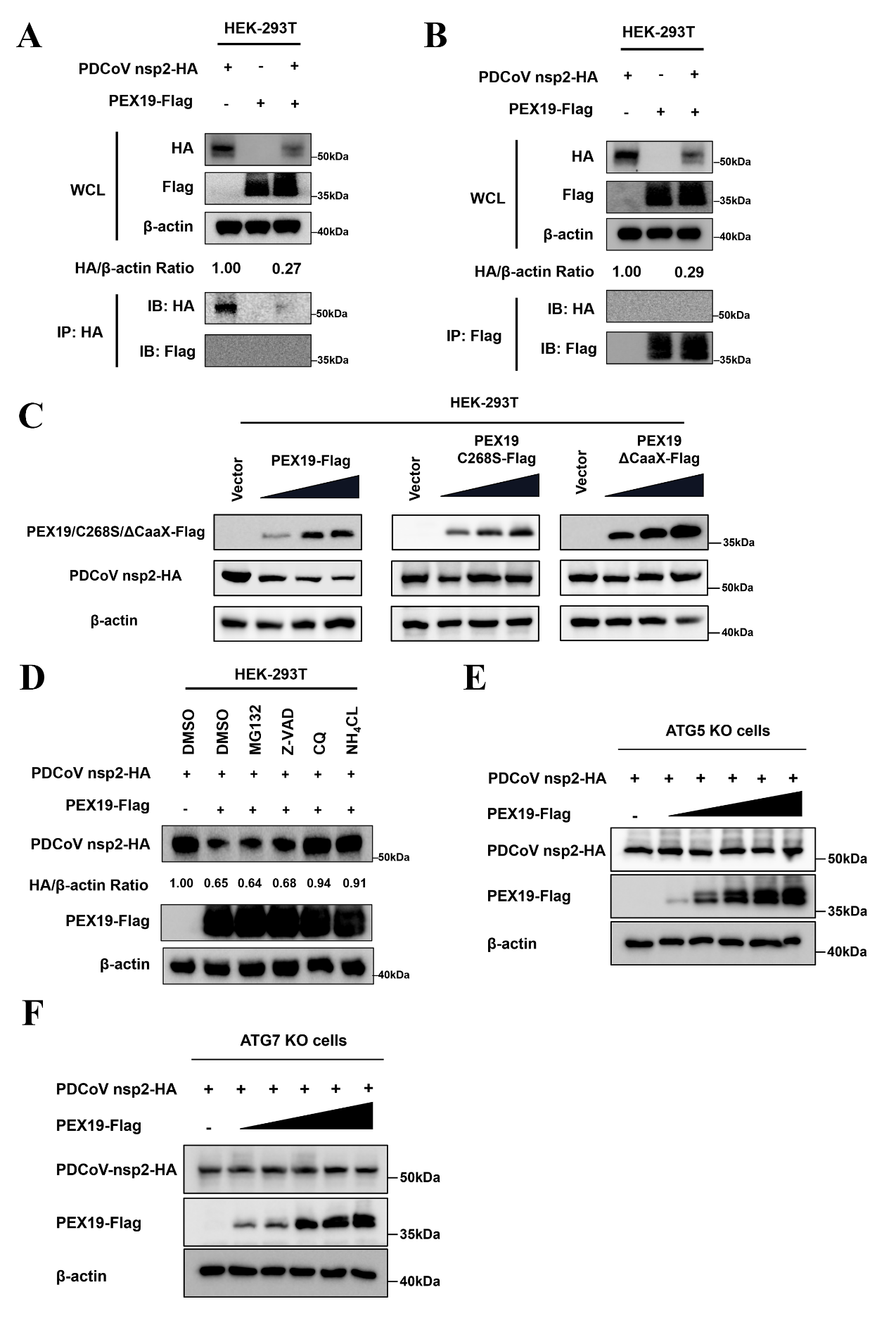
**

**Fig. S4. PEX19 promotes the degradation of PDCoV nsp2. (A, B)** PEX19 reduces the protein levels of PDCoV nsp2. HEK-293T cells were co-transfected with pCAGGS-Flag-PEX19 and pCAGGS-HA-nsp2. At 24 h post-transfection, cells were lysed, and the supernatants were collected. A small portion of each lysate was reserved as whole cell lysate (WCL), and the remaining lysates were subjected to coimmunoprecipitation (Co-IP) using anti-HA (A) or anti-Flag (B) monoclonal antibodies. **(C)** Farnesylation of PEX19 is required for the degradation of PDCoV nsp2. HEK-293T cells cultured in 6-well plates were co-transfected with 1 μg of pCAGGS-HA-nsp2 and increasing amounts (0, 0.25, 0.5, or 1.0 μg) of WT PEX19, PEX19 C268S, or PEX19 ΔCaaX constructs. Western blot analysis was performed using anti-HA, anti-Flag, and anti-β-actin antibodies. **(D)** Analysis of PEX19-mediated degradation pathway of PDCoV nsp2. HEK-293T cells were co-transfected with pCAGGS-HA-nsp2 and pCAGGS-Flag-PEX19 or empty vector. At 12 h post-transfection, cells were treated with DMSO, MG132 (10 μM), Z-VAD (20 μM), chloroquine (CQ, 5 μM), or NH₄Cl (10 mM) for an additional 12 h. Cell lysates were subsequently analyzed by Western blot. **(E, F)** PEX19-mediated degradation of PDCoV nsp2 is impaired in autophagy-deficient cells. HEK-293T cells with knockout of ATG5 (E) or ATG7 (F) were co-transfected with pCAGGS-HA-nsp2 and increasing amounts of pCAGGS-Flag-PEX19. At 24 h post-transfection, cells were harvested and lysates were analyzed by Western blot using anti-HA and anti-Flag antibodies to detect nsp2 and PEX19, respectively.

**Fig. S5**

**
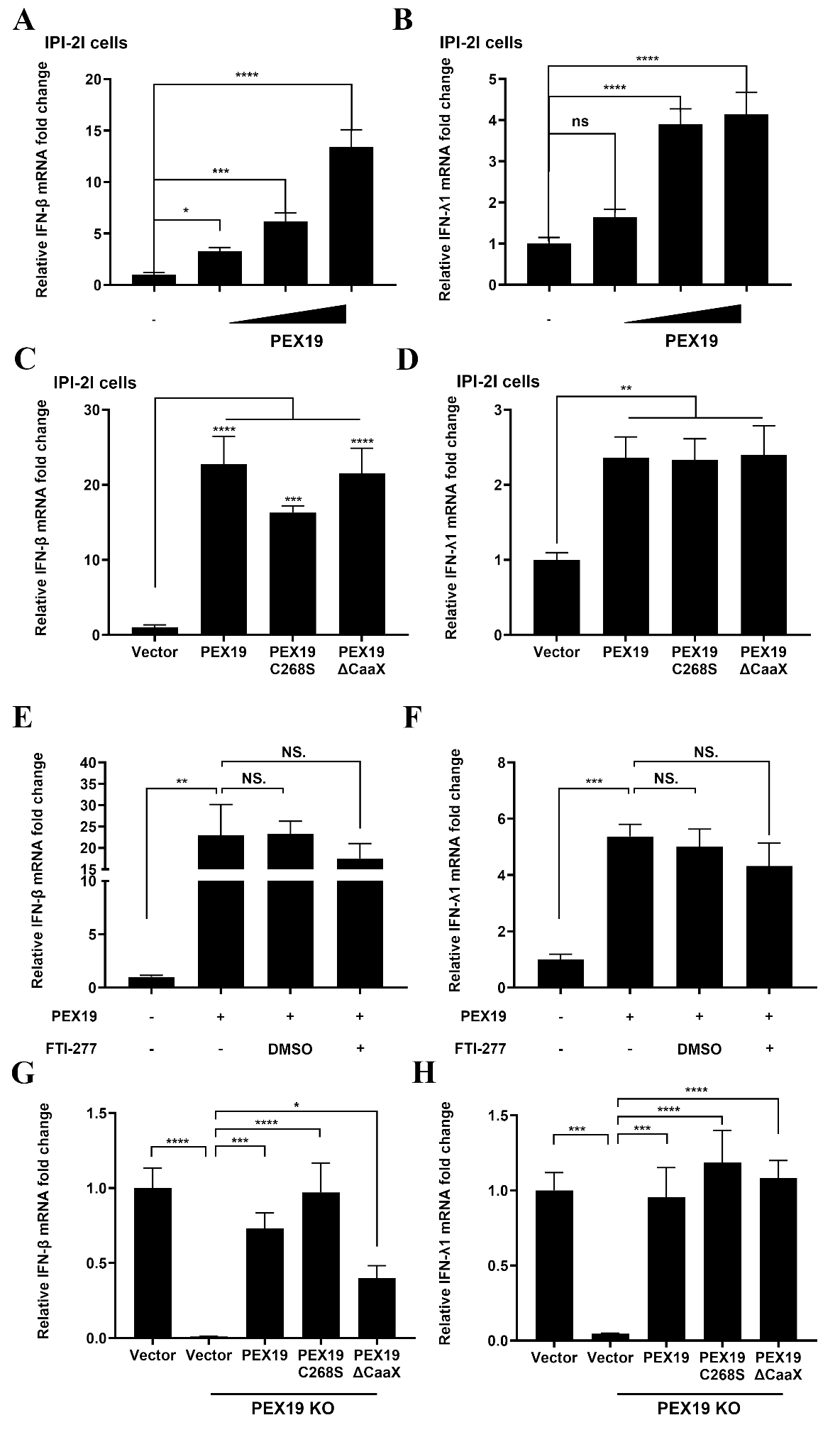
**

**Fig. S5. PEX19 induces low-level IFN responses. (A–B)** IFN induction by PEX19. IPI-2I cells were transfected with increasing amounts of pCAGGS-Flag-PEX19, and the mRNA levels of IFN-β (A) and IFN-λ1 (B) were determined by RT-qPCR at 24 h post-transfection. **(C–D)** Farnesylation-independent induction of IFN by PEX19. IPI-2I cells were transfected with empty vector, WT PEX19, PEX19 C268S, or PEX19 ΔCaaX constructs. The mRNA levels of IFN-β (E) and IFN-λ1 (F) were measured by RT-qPCR. **(E–F)** PEX19 promotes IFN production independently of farnesylation. LLC-PK1 cells were co-transfected with plasmids encoding HA-PDCoV nsp2 and Flag-PEX19. At 18 h post-transfection, cells were treated with 20 μM FTI-277 or DMSO (vehicle control) for 6 h. Total RNA was then extracted, and the mRNA levels of IFN-β and IFN-λ1 were quantified by RT-qPCR. **(G–H)** PEX19 promotes IFN-β and IFN-λ1 production during PDCoV infection independently of its farnesylation. LLC-PK1 WT cells or PEX19 KOcells were transfected with empty vector, WT PEX19, PEX19 C268S, or PEX19 ΔCaaX constructs. At 24 h post-transfection, cells were infected with PDCoV (MOI = 1) for 12 h. The mRNA levels of IFN-β and IFN-λ1 were quantified by RT-qPCR and normalized to GAPDH.


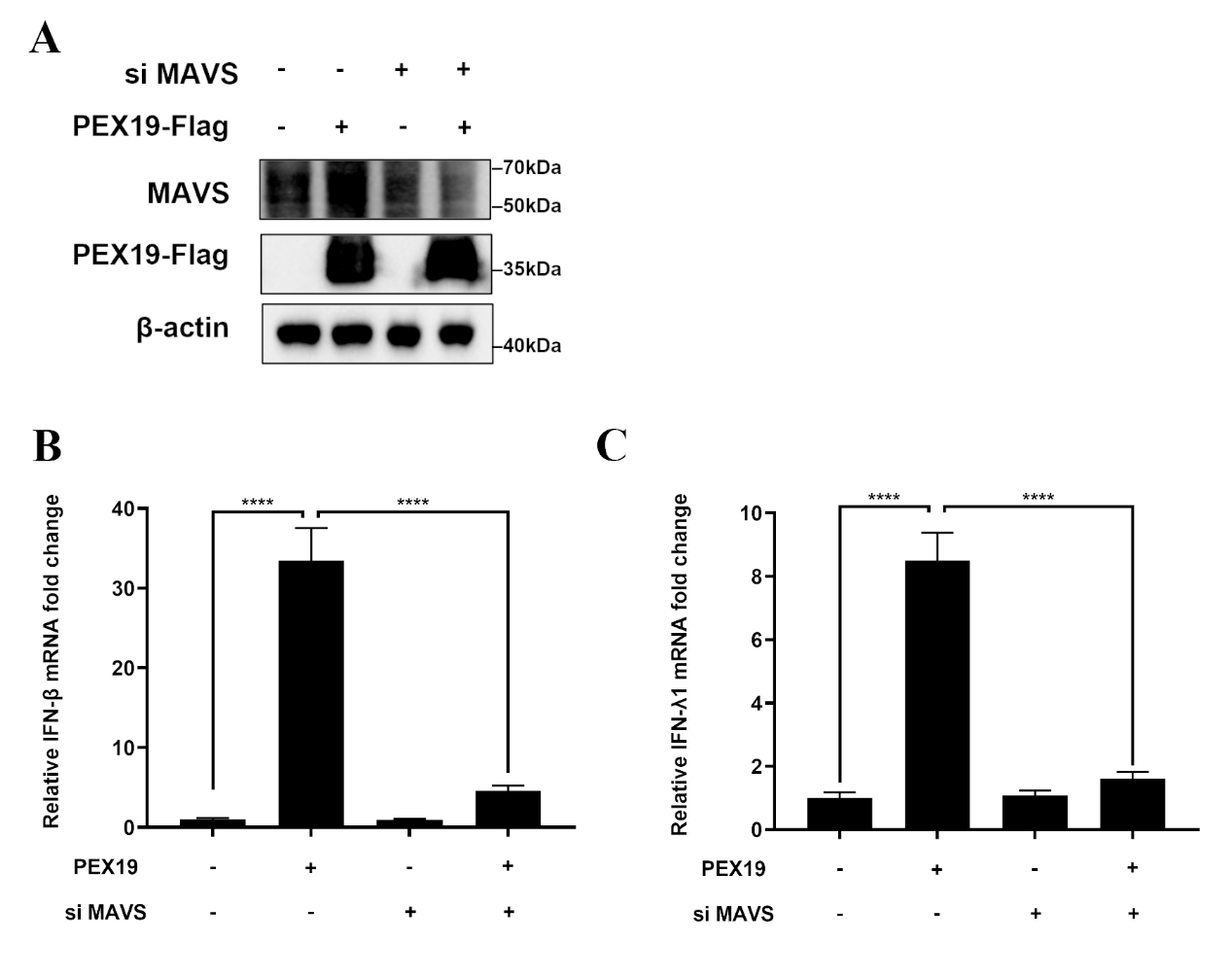
**Fig. S6**

**Fig. S6. PEX19 promotes IFN production via MAVS.** LLC-PK1 cells were initially transfected with control siRNA (siNC) or MAVS-targeting siRNA (siMAVS). At 24 h post-transfection, cells were further transfected with plasmids encoding WT PEX19 or an empty vector. After an additional 24 h, whole-cell lysates and total RNA were harvested for analysis. **(A)** Whole cell lysates were analyzed by Western blot to confirm MAVS knockdown efficiency. β actin served as a loading control. **(B–C)** Total RNA was extracted and subjected to RT‑qPCR to quantify the mRNA levels of IFN‑β (B) and IFN‑λ1 (C). Data are normalized to GAPDH.

**Fig. S7**


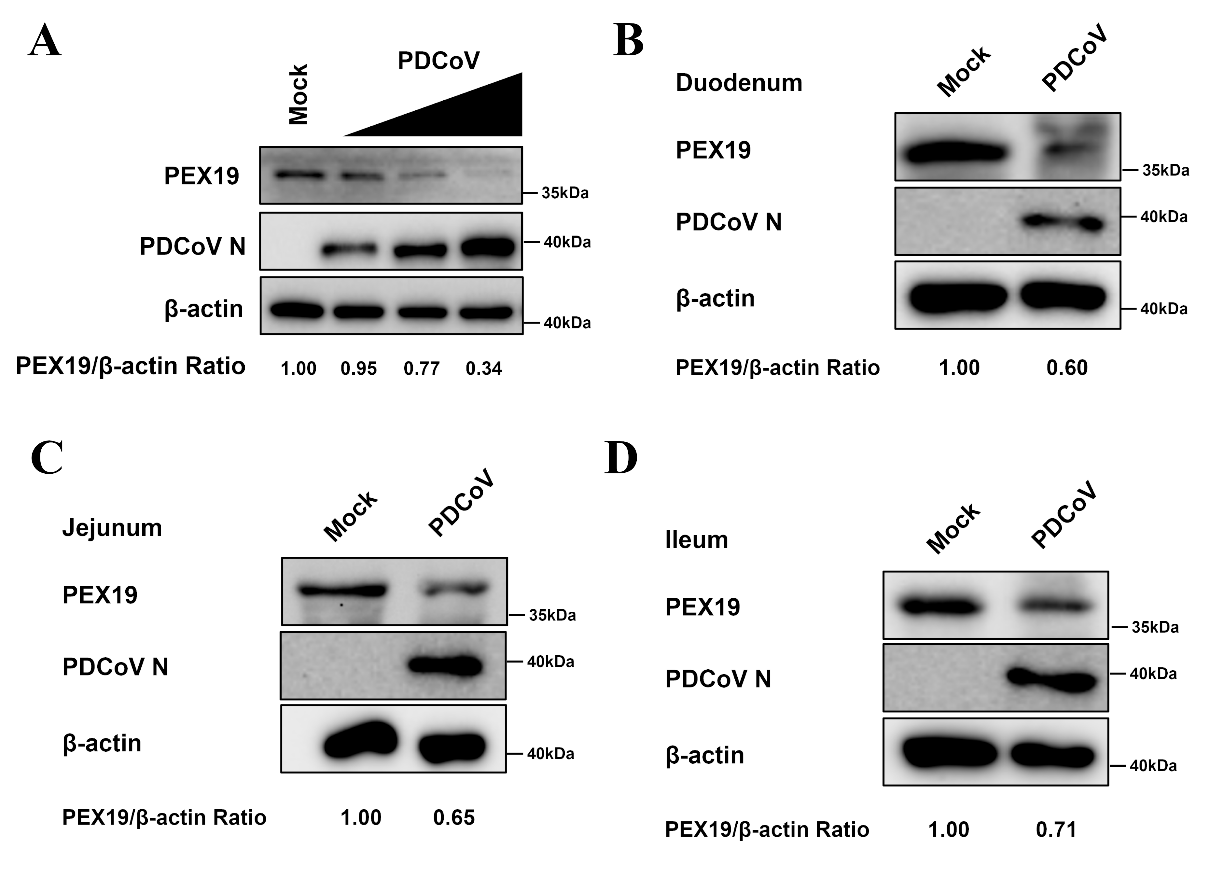


**Fig. S7.** **Effect of PDCoV infection on the expression of endogenous PEX19 protein.** PEX19 protein levels following PDCoV infection were examined by Western blot analysis. (**A**) LLC-PK1 cells were infected with PDCoV at increasing multiplicities of infection (MOIs) and harvested at 12 h post-infection. (**B-D**) Intestinal tissues from mock-infected and PDCoV-infected piglets were analyzed. β-actin was used as a loading control.
